# How many species of mammals are there in Brazil? New records of rare rodents (Rodentia: Cricetidae: Sigmodontinae) from Amazonia raise the current known diversity

**DOI:** 10.7717/peerj.4071

**Published:** 2017-12-15

**Authors:** Alexandre R. Percequillo, Jeronymo Dalapicolla, Edson F. Abreu-Júnior, Paulo Ricardo O. Roth, Katia M.P.M.B. Ferraz, Elisandra A. Chiquito

**Affiliations:** 1 Departamento de Ciências Biológicas, Escola Superior de Agricultura “Luiz de Queiroz”, Universidade de São Paulo, Piracicaba, São Paulo, Brazil; 2 Department of Life Sciences, The Natural History Museum, London, United Kingdon; 3 Department of Ecology and Evolutionary Biology, Museum of Zoology, University of Michigan, Ann Arbor, MI, United States of America; 4 Departamento de Ciências Florestais, Escola Superior de Agricultura “Luiz de Queiroz”, Universidade de São Paulo, Piracicaba, São Paulo, Brazil; 5 Departamento de Ciências Biológicas, Centro de Ciências Humanas e Naturais, Universidade Federal do Espírito Santo, Vitória, Espírito Santo, Brazil

**Keywords:** Mammalia, *Rhagomys*, *Neusticomys*, Neotropical region, Species distribution models, MaxEnt, randomForest, Molar occlusal topography, Skull morphology

## Abstract

**Background:**

Since 1996, when Vivo questioned how many species of mammals occur in Brazil, there has been a huge effort to assess this biodiversity. In this contribution, we present new records for rare species of the sigmodontine rodent genera *Rhagomys* and *Neusticomys* previously unknown to Brazilian Amazon. We provided detailed information on the morphologic variation to allow the proper identification of these species. We also furnished updated information on their collection, aiming to establish hypothesis of their geographic distribution, based on SDM’s, aiming to hypothesize potential occurrence areas for these species.

**Methods:**

Rodent specimens were sampled in separate inventories in two sites of Rondônia State (Hydroelectric Dam Jirau and Parque Nacional de Pacaás Novos) and one site in Pará State (Pacajá), Brazil, and were compared to specimens from museum collections to apply appropriate names. The SDM were conducted using two algorithms for rare species, MaxEnt and randomForest (RF), and were based on seven localities for *Rhagomys*, and 10 for *Neusticomys*.

**Results:**

All specimens were collected with pitfall traps. One specimen of genus *Rhagomys* was trapped in the Hydroelectric Dam Jirau. We identified this specimen as *R. longilingua*, and the SDM species indicates suitable areas for its occurrence at high elevations near on the Andes and lowlands of Amazon Basin to the South of the Rio Amazonas. Two specimens of *Neusticomys* were recorded, and we identified the specimen from Pacaás Novos as *N. peruviensis*, with SDM suggesting main areas of occurrence on Western Amazon. We applied the name *N. ferreirai* to the specimen from Pacajá, with SDM recovering suitable areas in Eastern Amazon.

**Discussion:**

We reinforced the importance of pitfall traps on the study of Neotropical rodents. We described morphologic variation within and among all species that do not invalidate their specific status, but in the near future a re-evaluation will be mandatory. The new records extended the species distribution considerably. SDM was successful to predict their distributions, as the two algorithms presented important differences in range size recovered by the models that can be explained by differences in the thresholds used for the construction of the models. Most suitable areas coincide with the areas facing most of the deforestation in Amazon. We added two rare species of sigmodontine rodents to the list of Brazilian Mammals, which now comprises 722 species (or 775 valid nominal taxa). Although more information is available than in 1996, it is essential that mammal experts maintain inventory and revisionary programs to update and revise this information. This is even more important, as changes in Brazilian environmental legislation are being discussed, suggesting reduced need for environmental impact reports prior to beginning commercial enterprises, resulting in the loss of information about native biodiversity in the affected areas.

## Introduction

How many species of mammals are there in Brazil? Vivo (1996) posed this question over 20 years ago, and mammalogists are still trying to answer it. The number of species of an area, whether a National Park, a country or a continent, depends upon several aspects, from conceptual issues—such as the current “*zeitgeist*” and species concept employed—to more practical ones—as the availability of specimens in collections (see Mora et al., 2011). In the last decade, several efforts had been made to assess the diversity of mammals in Brazil. Reis et al (2006, and 2011) edited volumes about “Mammals of Brazil”, focusing on reviews of the species diversity and biology. Fonseca et al. (1996) and later Paglia et al. (2012) produced annotated lists of the mammalian species of Brazil, listing 524 and 701 species, respectively. More recently, a coordinated effort of the Brazilian Society of Zoology (and its partner societies of ichthyology, herpetology, ornithology and mammalogy), along with the Brazilian government ministries, namely the Ministry of Environment and Ministry of Science and Technology, produced the Taxonomic Catalogue of the Fauna of Brazil (http://fauna.jbrj.gov.br/fauna/listaBrasil/ConsultaPublicaUC/ConsultaPublicaUC.do). This represents a strategic tool for the assessment of Brazilian diversity and the conservation of Brazilian fauna. In this catalogue, Percequillo & Gregorin (2017) recorded 720 species of mammals, an increment of 19 species in comparison to the last annotated list (Paglia et al., 2012). However, if one considers the valid taxa assigned to the category of subspecies, recognized in some groups (as bats, monkeys and rodents), the total number of valid nominal taxa is 773 (Percequillo & Gregorin, 2017), a consistent growth on the number of biological entities, regardless of their rank.

In the last two decades, several mammalian short and long-term inventories (sensu Voss & Emmons, 1996) had been conducted in Brazil (e.g., Abreu-Júnior et al., 2016; Abreu-Júnior, Brennand & Percequillo, 2017; see Bovendorp et al., 2017, for a review of inventories at the Brazilian Atlantic Forest). These include sampling performed through environmental impact assessments resultant of the Accelerated Growth Program of the Ministry of Planning from the Brazilian Government (since 2007; http://www.pac.gov.br) that fostered the national economic growth, through development of several infrastructure enterprises throughout Brazil. Reports of environmental impacts studies and environmental monitoring programs before, during, and after the construction as well as during the operation of these enterprises produced short and long-term mammal surveys (sensu Voss & Emmons, 1996), and hundreds of specimens were assembled and deposited at large and traditional, as well as small and new, Brazilian scientific collections. As a consequence of these efforts, new species (e.g., Bonvicino, Casado & Weksler, 2014; Moratelli & Dias, 2015; Pavan, 2015; Christoff et al., 2016; Pardiñas et al., 2016), and new distributional records throughout Brazilian states (e.g., Louzada et al., 2015; Abreu-Júnior et al., 2016; Rossi, Miranda & Semedo, 2016; Braga & Duda, 2017; Percequillo et al., 2017) have been produced, on a yearly basis. It is noteworthy, but not unexpected, that several of these novelties are from representatives of the Order Rodentia, mainly on the cricetid subfamily Sigmodontinae, one of the most speciose lineage of mammals in the world (D’Elía & Pardiñas, 2015). Recent compilations of South American mammal species (Gardner, 2008; Patton, Pardiñas & D’Elía, 2015) and the elaboration of the IUCN Red List of Threatened Species (IUCN, 2016) also have helped to build a more consistent scenario on the Brazilian Mammal diversity. Nevertheless, due to the vastness of Brazilian territory, there are still large gaps on the knowledge of diversity, including on this context not only the number of species, but also detailed information on morphology (diagnosis and geographic variation) and distribution (collection localities and hypothesized geographic distribution).

In this contribution, we present new records for rare species of the sigmodontine rodent genera *Rhagomys* and *Neusticomys* from the Brazilian Amazon, previously unknown to this country. A key issue on the establishment of the diversity is the proper identification of the species. This can be achieved through the employment of different traits, as morphology, karyology and gene sequences. As such, we intend to provide detailed information on the morphologic variation of the samples studied, to allow the proper identification of this rare entities and the assignment of the appropriate names to them. We also intend to furnish updated information on the collection localities of these species, aiming to establish hypothesis of their geographic distribution, based on species distribution models (SDM; Elith & Leathwick, 2009), aiming to hypothesize new potential occurrence areas for these species. Although poor datasets on collection localities makes SDM for rare species a complex task, often with temporal bias (samples were unevenly obtained through time, with old and more recent information assembled in the database) (Lomba et al., 2010), they are still achievable. Many studies have proposed different methods to generate good results from small samples (Pearson et al., 2007; Wisz et al., 2008; Shcheglovitova & Anderson, 2013; Breiner et al., 2015). Some authors contend that SDM for rare species should not to be considered as a prediction of the actual limits to the range of a species, but regions with similar environmental conditions to where the species may occur (e.g., Pearson et al., 2007), and here we follow this interpretation. We expect that the integration of these results on morphologic variation and species distribution will allow us to contribute to the knowledge on the diversity of sigmodontine rodents and consequently on the diversity and conservation of the Brazilian mammals.

## Materials & Methods

### Sampling

We perfomed independent field surveys at Hydroelectric Dam Jirau (UHE Jirau), 120 km upstream from Porto Velho (09°26′24″S, 64°50′24″W), Rondônia, in Rio Madeira, with elevations ranging from 85 to 281 m; at Parque Nacional de Pacaás Novos (10°46′57″S, 63°37′36″W), Rondônia, at elevations from 100 to 1,200 m; and at Pacajá, center-east of the state of Pará (0°02′06″S, 51°04′44″W), along an elevational range from 20 to 400 m. We performed a long-term sampling for the Program of Faunal Monitoring of UHE Jirau with conventional live-traps (Sherman and Tomahawk traps) and pitfall traps (as defined by Voss & Emmons, 1996), in three main sampling areas, named Caiçara, Mutum and Abunã; traps were baited with banana and a mixture of ground peanuts, oats, sardines and soybean oil. Each sampling area comprised four transects, totaling 12 transects (six on the left bank and six on the right bank of Rio Madeira). In each transect, five traplines—with six trap stations, each one including two traps on the ground and one trap about 2 m above the ground (totaling 90 traps per transect)—and five pitfall lines—each one with five 60 litres buckets connected by drift fences with 50 cm in height (totaling 25 buckets per transect) were set up. Sampling was conducted from February, 2010 to November, 2014, through four capture sessions per year (February, May, August, November) totaling 20 field surveys, 10 days long. The total sampling effort with conventional traps was 57,750 trap-nights and 7,602 pitfall-nights.

At the Parque Nacional de Pacaás Novos, a herpetology team coordinated by Dr. Miguel T. Rodrigues performed a short-term inventory (sensu Voss & Emmons, 1996) employing only pitfall traps, with 30 litres buckets arranged in stars (one central bucket with three buckets disposed in a radius of 4 m from the central one, all connected by drift fences about 40 cm in height). The sampling was conducted from April 9 to April 28, 2013, totaling an effort of 624 pitfall-nights.

We also conducted a short-term inventory of small mammals at Pacajá, Pará. Two surveys were performed for five consecutive days during dry (from October 22 to October 27, 2014) and wet (from January 22 to January 27, 2015) seasons. Conventional (Sherman and Tomahawk) and pitfall traps were employed. Eighty conventional traps were distributed in a transect with 40 trap stations (one Sherman and one Tomahawk per station) at 15 m intervals. Pitfall traps were arranged in six trap lines, each line consisting of six 60 litres buckets with drift fences about 50 cm in height; pitfall trap lines were set up perpendicularly to 900 m long transect, 150 m apart from each other. The total effort of conventional traps was 800 trap-nights and the pitfall traps effort was 300 pitfall-nights.

All specimens were trapped, sampled and prepared accordingly to the protocols established and approved by the Animal Care and Use Committee of the American Society of Mammalogists (Sikes & Animal Care and Use Committee of the American Society of Mammalogists, 2016), with approval from the Instituto Brasileiro do Meio Ambiente e dos Recursos Naturais Renováveis, Ministério do Meio Ambiente (260/2010, for UHE Jirau; 519/2014, for Pacajá) and from the Instituto Chico Mendes de Conservação da Biodiversidade, Ministério do Meio Ambiente (36753-3, for the Parque Nacional Pacaás Novos).

### Morphological analyses

We assessed qualitative and quantitative morphological traits in samples from UHE Jirau, Pacaás Novos and Pacajá, but also in specimens housed at the MZUSP (Museu de Zoologia da Universidade de São Paulo, São Paulo; includes the acronyms AB and EEB), MN (Museu Nacional da Universidade Federal do Rio de Janeiro, Rio de Janeiro), and BMNH (The Natural History Museum, London; see Data S1, for the list of specimens examined). For morphological traits nomenclature we followed Reig (1977), Carleton (1980), Voss (1988), and Luna & Patterson (2003). We follow the age classes defined by Voss (1988) for *Neusticomys* and Voss (1991) for *Rhagomys*.

External traits were measured by the collectors. We performed skull measurements (all measurements were performed by ARPercequillo) with a digital caliper (precision of 0.01 mm), based on the definitions provided by Voss (1988) and Voss (1991) for the genus *Neusticomys* and by Musser et al. (1998), Luna & Patterson (2003), and Villalpando, Vargas & Salazar-Bravo (2006) for the genus *Rhagomys*. The external and cranial measurements were: HBL, head and body length; LT, tail length; HF, hind foot length; Ear, ear length; Wt, body mass; GLS, greatest skull length; CIL, condylo-incisive length; OFL, orbital fossa length; LD, diastema length; LM1-3, upper molars series length; LM1-2, first two upper molars length; LIF, incisive foramen length; BIT, incisor tips breadth; BIF, incisive foramina breadth; BPB, bony palate breadth across first upper molars; PBB, palatal bridge breadth; LN, nasals length; BN, nasals breadth; BPL, bony palate length; PPL, post palatal length; LIB, least interorbital breadth; ZB, zygomatic breadth; BB, braincase breadth; BZP, zygomatic plate breadth; BM1, Ml breadth; HI, incisor height; DI, incisor depth; BOC, occipital condyles breadth.

### Species distribution models (SDM)

We performed SDM for two species of the genera *Neusticomys* (*N. ferreirai* with 7 localities and *N. peruviensis* with three localities) and for *Rhagomys longilingua* (with 7 localities), based on our new records and from occurrence records in the literature (see Results). We chose the two most commonly used algorithms for SDM of rare species: MaxEnt (version 3.3.3k; Phillips, Anderson & Schapire, 2006) and randomForest (RF) (Liaw & Wiener, 2002). For MaxEnt, we followed the method suggested by Shcheglovitova & Anderson (2013) using a jackknife approach, and controlling the model complexity. To estimate optimal model complexity, we used the ‘ENMeval’ package in R (Muscarella et al., 2014). We tested eight different feature class (FC) combinations (L, LQ, LQP, H, T, LQH, LQHP, LQHPT; where L, linear, Q, quadratic; H, hinge; P, product and T, threshold); considering regularization values (RM) from 0.5 to 3.0 (increments of 0.5). We used “n-1 jackknife” method for partitioning occurrence data. The models were built using these parameters: *R. longilingua* (FC: LQ; RM: 0.5); *N. ferreirai* (FC: LQ; RM: 0.5); *N. peruviensis* (FC: L; RM: 1.0) and the average model of seven replications to *R. longilingua*, seven to *N. ferreirai*, and three to *N. peruviensis,* constructed through the run type crossvalidate. For background points were chosen 10,000 points randomly, following the suggestion of Barbet-Massin et al. (2012). The models were built under the lowest presence threshold rule (LPT).

We used the algorithm RF from the R package “biomod2” (Thuiller et al., 2016). We employed the function “tuneRF” in the the R package “randomForest” (Liaw & Wiener, 2002) to calculate the number of variables randomly sampled at each split (mtry). The pseudo-absence points were separated into 20 replicates with 100 points each, randomly selected at a distance of two decimal degrees (around 250 km) from the points of presence, following Barbet-Massin et al. (2012). The final models were created using the average of the models with AUC greater than 0.7 and TSS greater than 0.7. The AUC and TSS, two metrics commonly used to evaluate model accuracy, were calculated for each algorithm for all species.

The same geographical area, encompassing the Amazon Basin to the south of Rio Amazonas, was applied to all species. Basin delimitations followed the HydroSheds (http://hydrosheds.cr.usgs.gov). We used 46 variables from remote sensing and interpolation methods to build the models (Data S1), as suggested by Wilson et al. (2013). The variables correspond to climate, land cover and topography, and we modeled for the present considering 30 arc seconds resolution (approximately 1 km^2^).

To choose the most important variables we followed Dormann et al. (2013). We performed a Principal Component Analysis (PCA) with the 46 variables, using the correlation matrix and standardized data. We analyzed the first 28 PCs which together account for more than 95% of the environmental variation. For each PC, we selected the variables that contributed more than 0.32 (10% of PC variance, according to Tabachnick & Fidell, 2007). Based on these selected variables, we performed a previous model in MaxEnt for each species with parameters as default. We calculated the importance of each variable to the model building using the jackknife method. In order to avoid overfitting the few occurrence points, we selected only the variables that contribute more than 5% to the previous models and that were not highly correlated with others variables (Data S2). We allowed a correlation coefficient between the variables of up to 0.7; this index was calculated in R (R Core Team, 2014).

## Results

### Species diversity: morphology and geographic distribution

Here, we present evidence to assign the samples obtained at the samplings above described to valid species, in order to provide information on their variation and geographic distribution. Based on such approach, we added two rare species of sigmodontine rodents to the list of Brazilian Mammals, which now comprises 722 species (or 775 valid nominal taxa).

### Genus *Rhagomys*

The conventional sampling effort in UHE Jirau comprised 57,750 trap-nights and obtained no specimens of *Rhagomys*. The only individual was sampled through pitfall traps, with an effort of 7,602 pitfall-nights. This specimen (MJ550) is a male with scrotal testes (age class 3), trapped on July 24, 2012, in a pitfall line set on the transition between “várzea” and “terra firme” forests, in Caiçara sampling site (transect 1, parcel 5), at an elevation of 105.1 m; it was preserved as skin, skull, carcass fixed in formalin (10%) and stored in alcohol (ethanol 70%), and hepatic tissue in ethanol (98%), stored at −20 °C.

**Figure 1:**
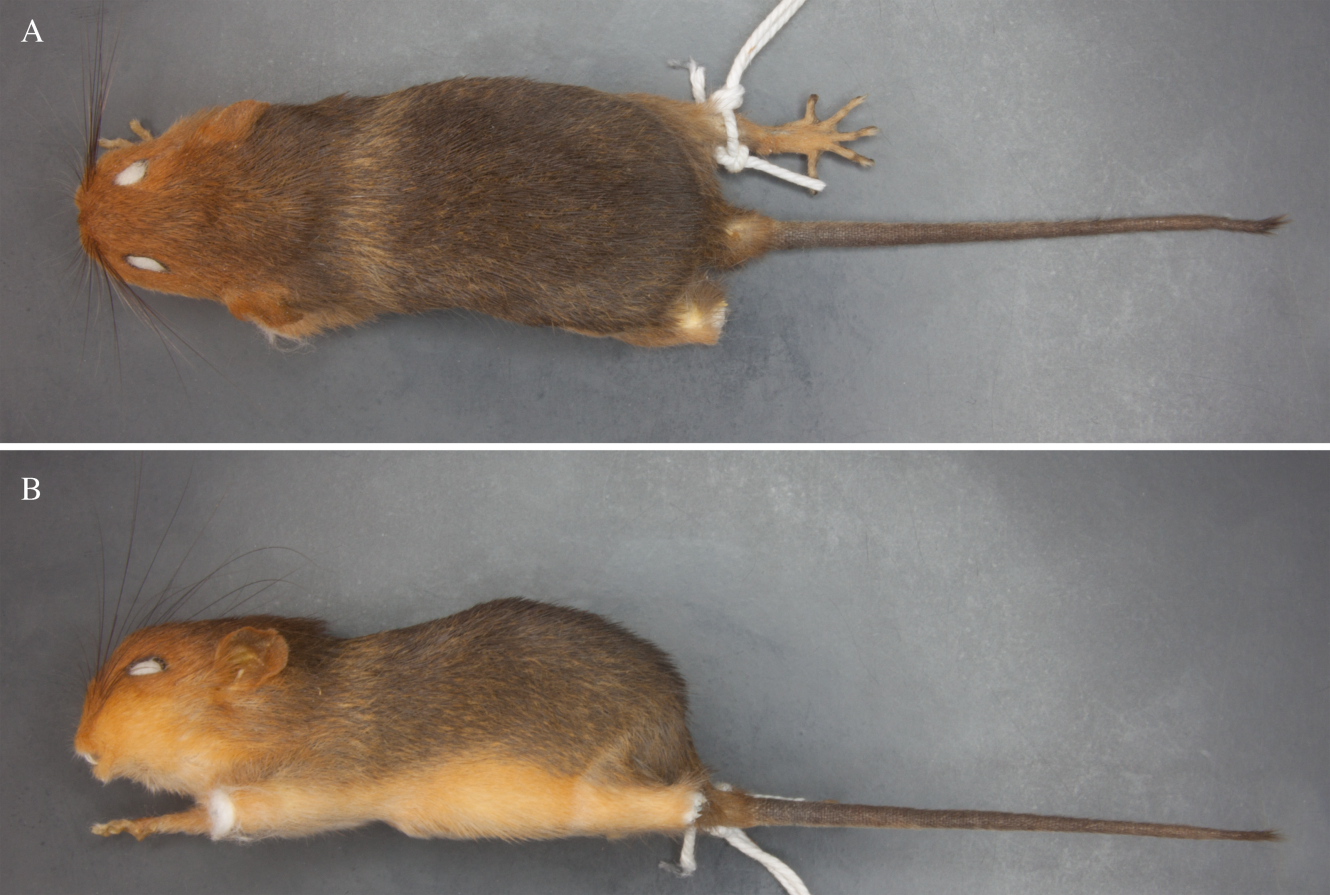
External morphology of *Rhagomys longilingua*. Taxidermized specimen of *Rhagomys longilingua*, MJ550, collected at Caiçara, UHE Jirau, Rondônia, Brazil, in dorsal (A) and lateral (B) views. Note the harsh and spiny pelage, the vivid orange head, the yellowish line behind the scapular region traversing all dorsal region, and the orangish venter.

MJ550 (Table 1; Fig. 1) presents short and spiny fur, with three kinds of hairs: viliforms, setiforms and aristiforms; the viliforms and aristiforms are regular hairs, while the setiforms are modified into spines. The viliforms are thin and wavy, banded, with a gray base, yellow or orange large subterminal band, and brown apical band; setiforms are large, wide and spiny, predominantly grayish-brown, and more brownish distally; and aristiforms are long and moderately wide, with gray base, long subterminal brown band and whitish apical band. The dorsal body color is brownish grossly grizzled with yellow; a yellowish line is visible behind the scapular region, traversing all dorsal region; the lateral region is more intensely grizzled with yellow, with an intense and vivid rusty-orange narrow band present on the latero-ventral portion of the body, from the lateral portion of the arm to the lateral portion of the leg; the venter is paler than lateral region, orange-buffy, with an orangish tinge prevalent on the abdominal region; the head is predominantly orangish, from the muzzle to the top of the head, immediately anteriorly to the ears; from the ears towards nape, the color is orange brown; ears are small and rounded, weakly covered by short hairs, orangish. Fore and hind feet are small (Table 1), wide and short; forefeet with digits II and V shorter, and digits III and IV longer, nearly equal in length, with short claws and short ungueal tufts; interdigital and tarsal pads fleshy; hindfeet with digit I very short, with distal portion enlarged, digits II, III and IV long, nearly equal in length, and digit V long, reaching the middle of the distal phalanx of digit IV; both fore and hindfeet weakly covered by short hairs, yellowish to orangish. The tail is as long as the head and body (Table 1), moderately hirsute, with a very short apical tuft (about 2 mm in length); the tail is weakly bicolored, being brownish above and grayish yellow below (on the basal half). The mystacial vibrissae are very long (ranging from 33 to 37 mm in length), surpassing ears when laid backwards.

**Table 1:** External and cranial measurements of two species genus *Rhagomys*, including the new record (MJ 550) and other known specimens of *R. longilingua* (CBF 7620 f, FMNH 170687 f, 175218 [BDP 4000] u, MUSM 17013 m; MUSA 13856 m, MUSA 13453 m, MUSA 15895 m; Luna & Patterson, 2003; Villalpando, Vargas & Salazar-Bravo, 2006;  Medina et al., 2017) and *R. rufescens* (AB 356 u, AB401 u, BMNH 86.2.8.5 f, CMUFLA 905 m, EEB849 m, F173 u, MN 65545 f, 66056 f, MZUSP 31952 u; Luna & Patterson, 2003; Percequillo, Gonçalves & Oliveira, 2004; Pinheiro, Hartmann & Geise, 2004, present study).

Measurements (mm; except Wt, grams)	*Rhagomys longilingua*			*Rhagomys* *rufescens*
	MJ550	Juveniles	Adults	Adults
HBL	71	52–80 (2)	89–103 (5)	65–107 (4)
LT	69	87–75 (2)	93–104 (5)	93–112 (4)
HF	17.5	16.1–13 (2)	17–20 (5)	19–21 (3)
Ear	11	10–9 (2)	12.1–14 (5)	12–15 (2)
Wt	16.5	21 (1)	24–25 (2)	12–32 (3)
GLS	25.77	22.31–25.34 (2)	27.72–28.58 (3)	23.68–28.5 (6)
CIL	23.17	19.97–22.99 (2)	25.02–26.01 (4)	21.01–28.52 (7)
OFL	9.64	8.29–9.69 (2)	10.5–10.72 (4)	8.99–10.95 (8)
LD	6.74	5.66–6.14 (2)	7.24–7.70 (4)	5.82–8.2 (9)
LM1–3	3.95	3.95–4.23 (2)	4.08–4.52 (4)	4.17–4.76 (8)
LIF	3.57	3.42–3.25 (2)	3.5–4.45 (4)	3.22–4.18 (9)
BIF	1.78	1.35–1.48 (2)	1.28–1.57 (2)	1.15–1.65 (9)
BPB	4.93	4.59–4.82 (2)	4.97–5.22 (4)	4.52–5.31 (4)
PBB	2.48	2–2.21 (2)	2.33–2.55 (2)	2.02–2.59 (5)
LN	8.89	5.91–7.8 (2)	8.51–8.84 (3)	7.09–9.39 (6)
BN	2.84	2.46 (1)	2.73–2.95 (3)	2.64–2.95 (3)
BPL	5.77	6.09 (1)	6.05–6.86 (4)	5.01–6.67 (8)
PPL	8.47	8.46 (1)	8.47–9.26 (4)	7.7–9.31 (3)
LIB	4.87	4.98–5.3 (2)	5.30–5.78 (4)	4.66–5.26 (9)
ZB	13.78	12.59–14.71 (2)	15.86–15.90 (4)	13.73–16.76 (6)
BB	12.37	12.01 (1)	11.24–13.89 (4)	12.81–13.63 (3)
BZP	2.94	2.18–2.75 (2)	2.96–3.3 (4)	2.32–4.35 (9)
BM1	1.08	1.1–1.21 (2)	1.15–1.3 (4)	1.12–1.31 (9)
DI	1.6	1.25–1.47 (2)	1.74–1.91 (4)	0.98–1.74 (5)

**Notes.**

Numbers furnished represent the observed range interval with sample size in parentheses.

Abbreviations for measurements as in text m male f female u sex undetermined

**Figure 2:**
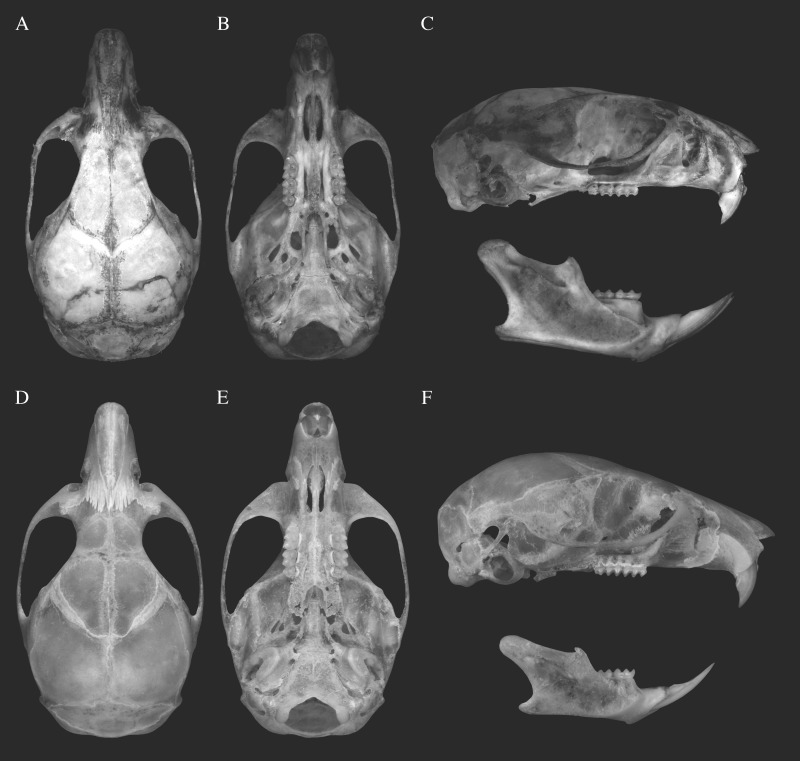
Skull morphology of species of genus *Rhagomys*. Plate with skulls of the genus *Rhagomys*: Skull of *Rhagomys longilingua* in dorsal (A), ventral (B) and lateral (C) views, MJ550, from Caiçara, UHE Jirau, Rondônia, Brazil; Skull of *Rhagomys rufescens* in dorsal (D), ventral (E) and lateral (F) views, EEB849, from Estação Ecológica de Bananal, São Paulo, Brazil. Note the presence of postorbital process, large fenestra on parapterygoid plates, sharp supraorbital margins, long and wide incisive foramina in *R. longilingua*.

The skull is small and gracile (Table 1; Fig. 2). The rostrum is narrow and short, with small and not inflated capsular process; the rostrum is also flanked by narrow and very shallow zygomatic notches. The lacrimal is large, and is in contact with both the frontal and maxillary bones. The interorbital region is narrow (Table 1), with squared and slightly beaded supraorbital margins divergent posteriorly; the supraorbital margins are continuous with squared temporal margins (ridges are not present). A delicate postorbital crest is present and visible dorsally, along the sutures of frontal and squamosal. The zygomatic arches are narrow and not very projected laterally, being mostly parallel. The braincase is elongated, with a more rounded profile; the lambdoidal crests are weakly developed and the occipital and nuchal ridges or crests are missing (these regions are not even squared, as is common in other sigmodontines). The interparietal is wide and very long, contributing to the long and round profile of the braincase. In lateral view, the zygomatic plate is very narrow (Fig. 2), with free dorsal margin very short; the anterior margin is almost straight, slightly sloping posteriorly. The zygomatic arch is narrow, and the jugal is narrow and delicate, but long, and the maxillary and squamosal roots are not overlapped. The alisphenoid strut is very robust, defining discrete and independent oval accessory and buccinador-masticatory foramina. The squamoso-alisphenoid groove is absent, the anastomotic channel is present, and the stapedial foramen is absent, configuring the derived stapedial and carotid circulatory pattern (III). The suspensory process of squamosal is present, although small, and overlapped to a small and delicate tegmen tympanii, configuring a small postglenoid foramen; the subsquamosal fenestra is wide. The auditory meatus is wide; the mastoid is rounded, and without fenestra. The incisive foramina are short and narrow (Table 1), with anterior and posterior margins rounded and lateral margins wider medially (lateral margins convex); the posterior margin is distant from the molar series. The palate is long and wide (Table 1), with simple and small posterolateral palatal pits. The mesopterygoid fossa is narrow, with rounded anterior margin; the anterior margin is distant from the molar series, the roof of mesopterygoid fossa is completely ossified, without vacuities. The parapterygoid fossae are narrow, with similar width to the mesopterygoid fossa; the fossae are perforated by large and ovate fenestra, and by moderate posterior opening of the alisphenoid canal. The foramen lacerum medium is very narrow, as the auditory bulla is closely positioned to the parapterygoid fossa. The auditory bulla is flask shaped, with Eustachian tube wide and short.

The mandible is unique, with a flat ventral surface; the mandible ramus is low. The condyloid process is developed and projected posteriorly; the coronoid process is small and triangular, lower than the condyloid process; the angular process is short, much anterior than the condyloid process; the inferior and superior notches are shallow. The capsular process of the lower incisor is well developed and positioned near the condyloid process. The retromolar fossa is wide and deep.

**Figure 3:**
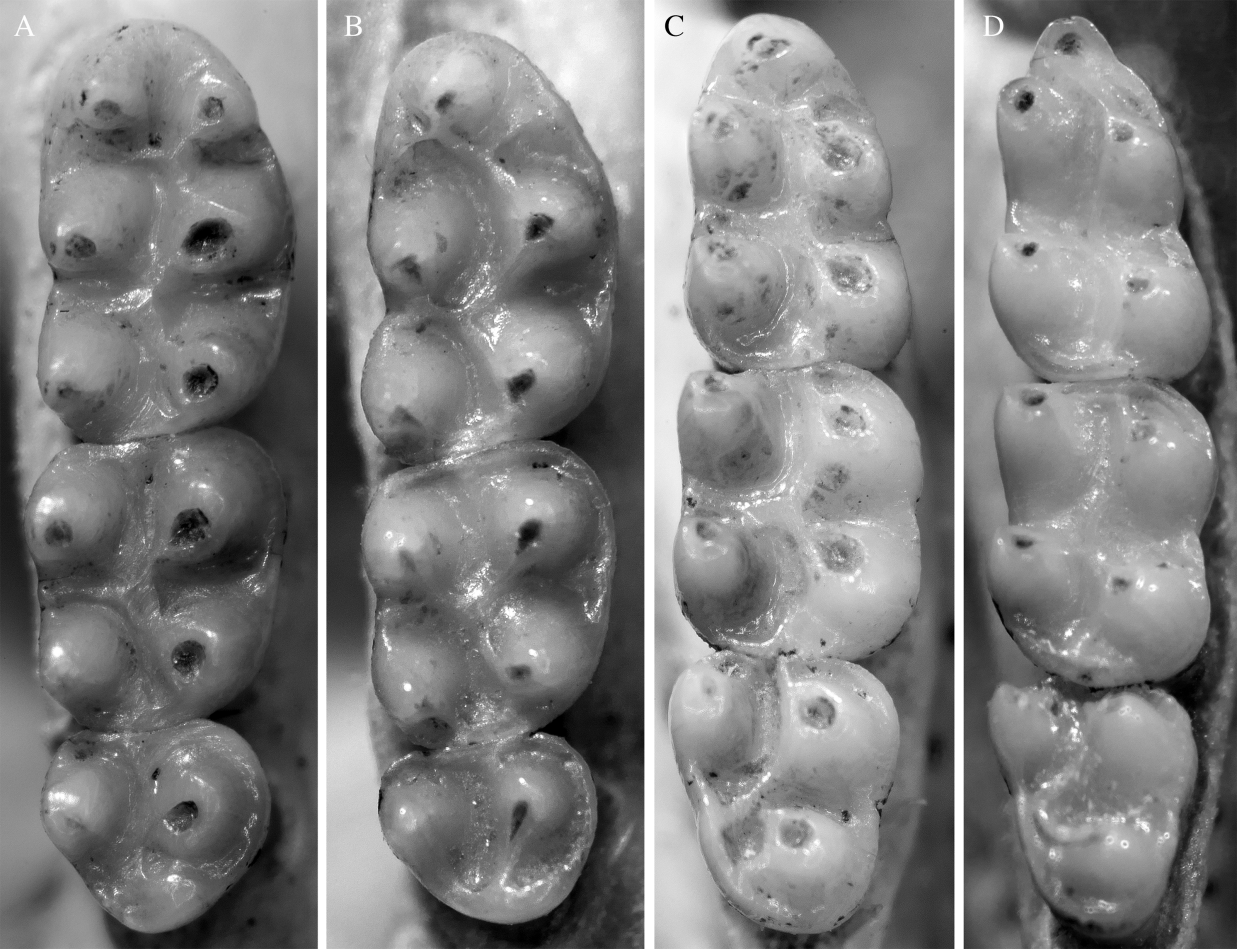
Molar morphology of species of *Rhagomys*. Plate with molar series of the species of genus *Rhagomys*: Right upper series of *R. rufescens*, AB401, from Ribeirão Grande, São Paulo, Brazil (A); right upper series of *R. longilingua*, MJ550 (B); right lower series of *R. rufescens*, AB401 (C); right upper series of *R. longilingua*, MJ550 (D). Note the general similarity between both species, but also note the absence of the anterolingual conule, the reduced procingulum of the m1, the absence of the posterocrista in m1 and m3 of *R. longilingua*.

Upper molars series are small and delicate (Table 1; Fig. 3); these series are slightly convergent anteriorly; the four main cusps are the most visible structures on molar surface; the cusps are arranged in alternate pairs, the lingual positioned anteriorly than the labial ones; occlusal surface of molars are predominantly formed by enamel, as exposition of dentine is restricted to the apical portion of the main cusps; labial and lingual flexi are shallow not quite conspicuous, the lingual ones more discernible than the labial. M1 with very narrow procingulum, as the lingual anteroconule is absent, as well as the anteromedian crista. The anteroloph is small, but present, reaching the labial surface. The paracone and protocone are connected posteromedially to the median mure, as well as the mesoloph, that is narrow and delicate, and reach the labial margin. The posteroloph is present. M2 is quite similar to M1. M3 very small, with paracone and protocone present; the posterior cones (metacone and hypocone), as well their associated structures are absent. Lower molars also with main cusps arranged in alternate pairs, the lingual anteriorly positioned to the labial; flexids are also less evident. The m1 exhibits a very small procingulum, medially positioned and without conspicuous flexid. The mesolophid is absent and the posterocrista is present. The m2 is very similar to m1. The m3 is also similar to m2, but the entoconid is quite reduced, being a small protuberance on the lingual margin.

This specimen shares with the holotype and with the original description of *R. longilingua* (Luna & Patterson, 2003) the presence of hairs transformed into spines; the predominance of orange color of the head; the presence of large and ovate fenestrae on the parapterygoid fossae; the presence of well-developed postorbital process; and the presence of a large retromolar fossa. Regarding quantitative traits, the Brazilian specimen is similar in size to the Bolivian (Villalpando, Vargas & Salazar-Bravo, 2006) and the Peruvian specimens (Luna & Patterson, 2003;  Medina et al., 2017), with values within the observed range (Table 1). Nevertheless, there are some differences, with the specimen from Rondônia exhibiting slightly longer rostrum, wider incisive foramina, narrower interorbital region and narrower first upper molar. Therefore, based on these qualitative and quantitative evidences, we hypothesize that this specimen of *Rhagomys* from UHE Jirau belongs to the species *Rhagomys longilingua* (Luna & Patterson, 2003), being the first record of this species for Brazil.

**Figure 4:**
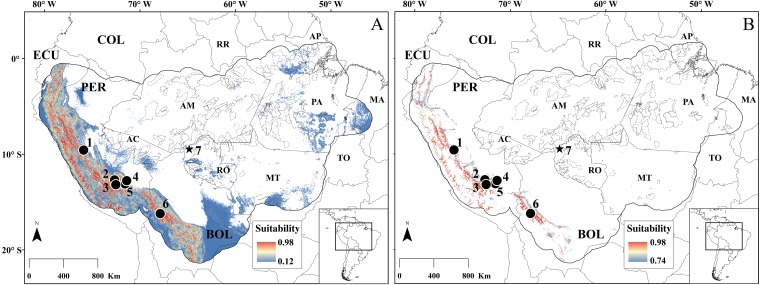
SDM for *Rhagomys longilingua*. Species distribution models for *R. longilingua* built with MaxEnt (A) and RF (B). Dots represent the points used in building the model, and the star is the new occurrence point reported in this study. Continuous gray line represent the political limits, continuous black line represent the modeled area (see text for explanation), and the dotted black line represent the limits of Brazilian conservation units (on federal, state and municipal levels). Minimum and maximum suitability values are represented by different colors. Localities numbers are listed in Table 2. Acronyms: in larger captions, the South American countries: BOL, Bolívia; COL, Colombia; EC, Ecuador; PER, Peru; in smaller captions the Brazilian states: AC, Acre; AM, Amazonas; AP, Amapá; MA, Maranhão; MT, Mato Grosso; PA, Pará; RO, Rondônia; RR, Roraima.

The SDM (Table 2 for data on samples employed in SDM analysis) for *Rhagomys longilingua* showed different results depending on the algorithm used. The two performance metrics of SDM were satisfactory for RF (AUC 0.994; TSS: 0.984), whereas for MaxEnt only AUC performed well (AUC: 0.915; TSS: 0.525). The differences persisted when comparing the areas recovered in the SDM as suitable for the occurrence of *R. longilingua*. The area predicted by the MaxEnt (Fig. 4A) is large (1,451,280.65 km^2^), and included, in addition to the Andes, much of the Amazon Basin to the south of the Rio Amazonas, whereas the area predicted by the RF (Fig. 4B) (143,642.24 km^2^) is restricted to the high elevations near the Andes. Thus, only MaxEnt recovered areas close to the new occurrence record as suitable for this species. The most important variable for the construction of both models was a topographic variable (OPISRE: 0.98 for MaxEnt and 0.56 for RF) (see Data S2 for importance of the other variables). Regardless sampling limitations, our models recovered potential occurrence areas for *R. longilingua* along the Andes foothills, with high suitability, and throughout the south portion of Amazon basin, mostly with lower suitability, but an intermediate to high suitability was detected in the southeastern portion of Pará State.

**Table 2:** Localities employed for species distribution models (SDM) with their respective sources. The site number corresponds to the points in maps of Fig. 4 for *Rhagomys longilingua* and of Fig. 8 for *Neusticomys ferreirai* and *N. peruviensis*. The elevation values for the localities were extracted from the variable altitude from WorldClim with resolution of 30 arc seconds when they were not informed by the source.

Species	Site	Longitude (decimals)	Latitude (decimals)	Elevation (m)	Locality	Source
*Rhagomys longilingua*	1	−75.903	−9.575	936	Santa Rita Alta, Chaglla, Huánuco	Medina et al. (2017)
	2	−72.663	−12.690	2,155	Urusayhua Mountain, Echarate, Cusco	Medina et al. (2017)
	3	−72.532	−13.187	2,200	Wiñaywayna Biological Station, Machu Picchu Historic Sanctuary	Medina et al. (2017)
	4	−71.390	−12.770	468	Maskoitania, Río Alto Madre de Dios, Peru	Luna & Patterson (2003)
	5	−71.569	−13.105	2,037	Manu Biosphere Reserve, Cuzco, Peru	Luna & Patterson (2003)
	6	−67.887	−16.213	1,989	Bajo Hornuni, Parque Nacional y Area Natural de Manejo Integrado Cotapata, Bolivia	Villalpando, Vargas & Salazar-Bravo (2006)
	**7**	−**64.840**	−**9.440**	**105.1**	**UHE Jirau, Porto Velho, Rondônia, Brazil**	**This study**
*Neusticomys peruviensis*	8	−71.283	−11.950	283	Pakitza, Madre de Dios, Peru	Pacheco & Vivar (1996)*apud*Voss (2015)
	9	−71.217	−10.133	445	Balta, Río Curanja, Ucayali, Peru	Musser & Gardner (1974)*apud*Voss (2015)
	**10**	−**63.627**	−**10.783**	**393**	**Parque Nacional de Pacaás Novos, Rondônia, Brazil**	**This study**
*Neusticomys ferreirai*	11	−59.450	−10.167	240	Aripuanã, Mato Grosso, Brazil	Miranda et al. (2012)
	12	−58.483	−10.233	81	Juruena, Mato Grosso, Brazil	Percequillo, Carmignotto & Silva (2005)
	13	−56.542	−4.652	109	Parque Nacional da Amazônia, Pará, Brazil	Oliveira et al. (2016)
	14	−51.938	−3.584	180	Senador José Porfírio, Pará, Brazil	Braga & Duda (2017)
	**15**	−**51.079**	−**4.035**	**223**	**Pacajá, Pará, Brazil**	**This study**
	16	−50.550	−5.767	684	Floresta Nacional Tapirapé-Aquiri, Pará, Brazil	Miranda et al. (2012)
	17	−50.250	−6.050	105	Floresta Nacional dos Carajás, Pará, Brazil	Braga & Duda (2017)

### Genus *Neusticomys*

We obtained two specimens of the genus *Neusticomys*, one from Pacaás Novos, Rondônia and other from the sampling in Pacajá, Pará. The specimen of *Neusticomys* from Pacaás Novos, MTR25579, is an adult male with scrotal testes (age class 4/f/a), captured on April 13, 2013, in a pitfall trap placed on a flat lowland “terra firme” forest, about 300 m from a large stream (10 m wide, near the capture site), at an elevation of 393 m. MTR25579 was fixed in formalin (10%) and stored in alcohol (ethanol 70%), and also present a removed and cleaned skull, and hepatic tissue in ethanol (98%), stored at −20 °C.

**Figure 5:**
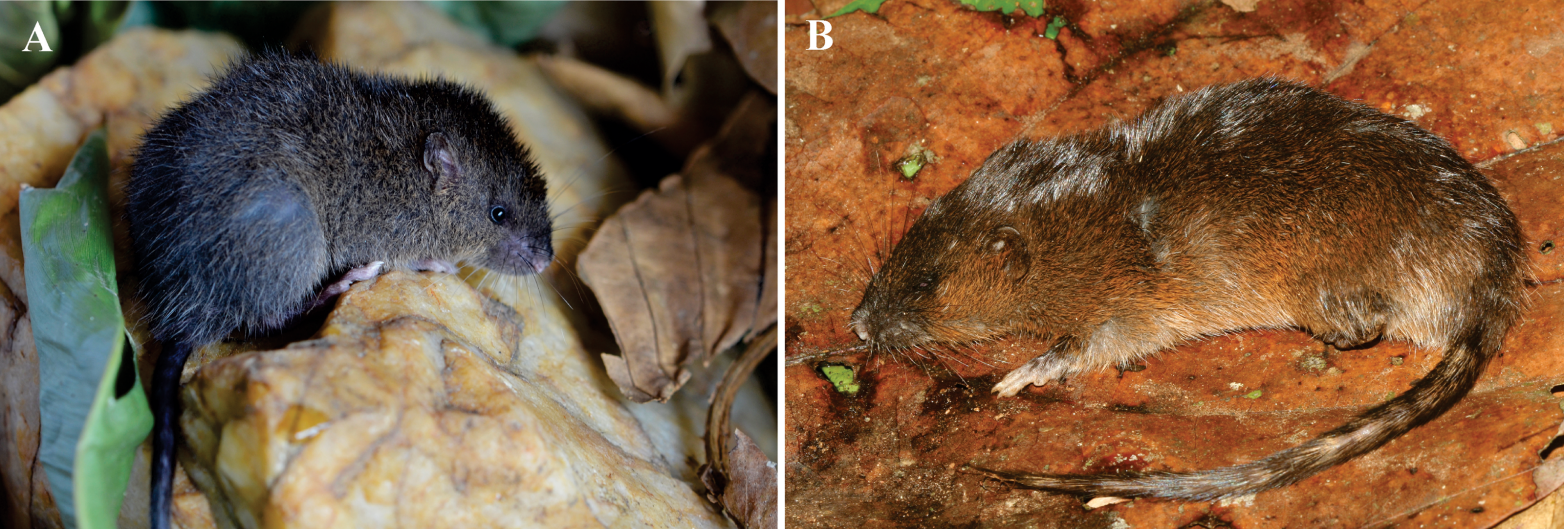
External morphology of species of genus *Neusticomys*. Specimens of the genus *Neusticomys*: Live specimen of *N. ferreirai*, X1M27, captured at Pacajá, Pará, Brazil (A); recently killed specimen of *N. peruviensis*, MTR25579, captured at Pacaás Novos, Rondônia, Brazil (B). Note the orange/ferruginous color of *N. peruviensis*, in comparison to the brownish hues of *N. ferreirai*; note the small eyes, small pinnae and dense mystacial vibrissae of both species.

MTR25579 (Table 3; Fig. 5) presents a dense and soft fur, with three kinds of hairs: viliforms, setiforms and aristiforms. The viliforms are thin and wavy, banded, with a gray base, orange copper large subterminal band, and brown apical band; setiforms are large, with a gray base and orange copper large subterminal band, and brown apical band, or with gray base and brownish distally; and aristiforms are long and moderately wide, with gray base, long subterminal brown band and whitish apical band. The dorsal body color is copper orange intensely and finely grizzled with brown; an intense copperish hue is present on the lateral portion of the body, from the lateral portion of the arm to the lateral portion of the leg; this coppery color is also present on the ventral region, and the transition from lateral to ventral portions is subtle. The head is similar to the dorsal pelage, except from the muzzle that is predominantly brown; ears are small and rounded (Table 3), almost concealed by the fur, densely covered by moderately long hairs on the internal surface and on the external margin; internal hairs are orangish, with apical brown band and external hairs are brown. Fore and hind feet are small (Table 3), wide and short; forefeet with digits II and V shorter, and digits III and IV longer, nearly equal in length, with short claws and very short ungueal tufts; interdigital and tarsal pads fleshy; hindfeet with digit I very short, digits II, III and IV long, nearly equal in length (digit III slightly longer), and digit V long, reaching the middle of the distal phalanx of digit IV; both fore and hindfeet weakly covered by short hairs, with brown base and orange/ochre apex; the ungual tufts are very sparse and short; interdigital pads and thernar pad are fleshy, hypothenar pad absent. The tail is as long as the head and body (Table 3), densely hirsute, with a long apical tuft (hairs ranging from 10 to 17 mm in length); the tail is unicolored, brownish, with some apical hairs unpigmented. The mystacial vibrissae are dense and long, slightly surpassing ears when laid backwards.

**Table 3:** External and cranial measurements of five species genus *Neusticomys*, including the new records: *N. ferreirai* (MZUSP 32092 f, 32093 m; X1M27 f), *N. mussoi* (CVULA-I-1637 f, 1638 m; Ochoa & Soriano, 1991), *N. oyapocki* (MNHN 1977-775 m, 1995.3234 m, AMNH 267597 m, MPEG 34251 f; Voss, 1988; Voss, Lunde & Simmons, 2001; Nunes, 2002), *N. peruviensis* (LSU 14407 m, MTR25579 m; Voss, 1988), *N. venezuelae* (AMNH 69907 m, 69908 f, 257344 f, 257345 m, USNM 406123 f, EBRG 15951 u, 15973 u; Voss, 1988; Ochoa & Soriano, 1991; Voss, Lunde & Simmons, 2001).

Measurements (mm; except Wt, grams)	*Neusticomys* *ferreirai*		*Neusticomysmussoi*	*Neusticomysoyapocki*	*Neusticomys* *peruviensis*		*Neusticomys* venezuelae
	X1M27	Type specimens			MTR 25579		
BL	108	106–105 (2)	94–118 (2)	102–114 (4)	–	128	100–132 (7)
LT	59	85–79 (2)	–	66–87 (4)	–	108	105–120 (5)
HF	22/23.5	21/22.5–22/24 (2)	21 (2)	23–26 (4)	–	30	25–28 (7)
Ear	12	11–11.5 (2)	10 (2)	6–12 (4)	–	12	10–13 (7)
Wt	24	34–25 (2)	–	21–47 (3)	–	–	58–66 (2)
CIL	23.89	24.5–24.6 (2)	24.1–24.7 (2)	24–27.9 (4)	26.87	28.1	26.0–29.3 (6)
LD	6.49	6.7–6.9 (2)	6.2 (2)	6.7–7.8 (4)	7.56	7.5	6.7–7.6 (7)
LM1-3	–	3.5–3.2 (2)	3.3–3.4 (2)	–	3.32	3.8	4.0–4.3 (7)
LM1-2	2.92	3–2.8 (2)	–	2.9–3.0 (4)	2.61	3.2	3.3–3.6 (5)
LIF	4.19	5–4.6 (2)	4.6–4.8 (2)	4.1–5.2 (4)	4.85	5.6	4.8–5.7 (6)
BIT	1.52	1.8–1.8 (2)	1.6–1.9 (2)	1.9 (1)	1.94	2.2	1.5–2.0 (6)
BIF	1.86	1.9–2 (2)	1.8 (2)	2.3 (1)	2.17	2.0	1.8–2.2 (6)
PBB	2.54	2.6–2.6 (2)	2.5–2.6 (2)	2.5–3.0 (4)	2.9	3.3	2.5–3.1 (6)
LN	9.39	9.7–9.8 (2)	9.5 (1)	–	10.69	11.1	8.8–12.3 (6)
BN	3.65	3.1–3.3 (2)	3.3 (2)	3.3 (1)	3.77	3.7	3.2–3.6 (6)
LIB	5.37	4.9–4.8 (2)	4.5–4.6 (2)	4.8–5.4 (4)	5.02	5.2	5.0–5.4 (6)
ZB	12.86	13–12.6 (2)	12.2–12.3 (2)	12.3–15.1 (4)	14.00	14.4	12.8–14.5 (5)
BB	11.40	11.2–10.9 (2)	11.0 (2)	11.2 (1)	10.57	12.8	11.6–13.0 (6)
BZP	1.30	1.4–1.4 (2)	1.1 (2)	1.4–1.5 (4)	1.44	1.3	1.1–1.5 (7)
BM1	1.08	1.3–1.2 (2)	1.2 (2)	1.1 (4)	1.30	1.3	1.3–1.5 (7)
HI	3.78	4.3–4.4 (2)	4.1–4.3 (2)	5.2 (1)	4.79	4.9	4.0–5.1 (6)
DI	1.34	1.4–1.6 (2)	1.5 (2)	1.6 (1)	1.67	1.9	1.2–1.8 (6)
BOC	6.86	6.8–6.9 (2)	6.2–6.5 (2)	7.1 (1)	7.09	7.6	7.1–7.6 (5)

**Notes.**

Numbers furnished represent the observed range interval with sample size in parentheses.

Abbreviations for measurements as in text m male f female u sex undetermined

**Figure 6:**
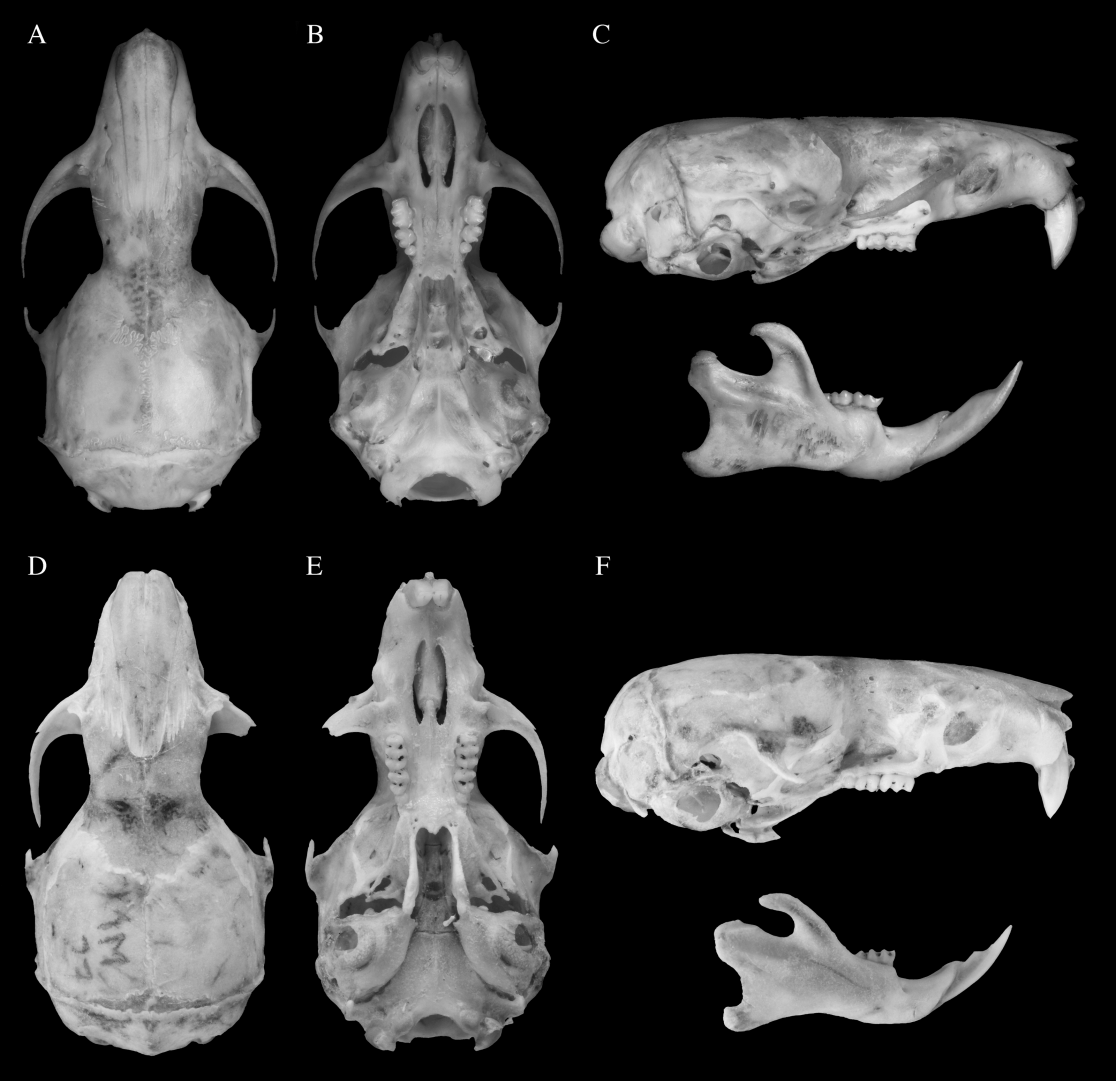
Skulls of the species of genus *Neusticomys*. Plate with skulls of the genus *Neusticomys*: Skull of *N. peruviensis* in dorsal (A), ventral (B) and lateral (C) views, MTR25579, from Pacaás Novos, Rondônia, Brazil; skull of *N. ferreirai* in dorsal (D), ventral (E) and lateral (F) views, X1M27, from Pacajá, Pará, Brazil. Note the broader interorbital region and larger auditory bullae of *R. ferreirai*.

The skull (Table 3; Fig. 6) is moderately robust. The rostrum is wide and short, strongly divergent posteriorly, with nasolacrimal capsular process not visible; the rostrum lacks zygomatic notches. Nasals and premaxillaries well projected posteriorly, largely surpassing the fronto-maxillar suture and participating on the interorbital region. The lacrimal is moderately large, and is in contact only with the maxillary bone. The interorbital region is narrow (Table 3), with rounded supraorbital margins, strongly convergent posteriorly, with a distinct “bottleneck” in its narrower portion; temporal margins are absent. A discrete postorbital crest is present and visible dorsally, along the sutures of frontal and squamosal bones. The zygomatic arches are narrow and incomplete, not projected laterally, and mostly parallel. The braincase is elongate, with a rounded profile, as the dorsal and lateral surfaces are not clearly delimited; the lambdoidal crests are moderately developed and occipital and nuchal regions are weakly pronounced. The interparietal is short and narrow. The occipital condyles are quite projected posteriorly, being noticeably visible dorsally. In lateral view, the rostrum appears long; the gnathic process is long and robust, well project beyond the anterior surface on incisors. The zygomatic plate is very narrow (Table 3), without free dorsal margin; the anterior margin is concave, with a distinct masseteric tubercule on the ventral portion of the plate. The zygomatic arch is very narrow and incomplete, and the jugal is absent. The alisphenoid strut is very robust, defining discrete and independent oval accessory and buccinador-masticatory foramina. The squamoso-alisphenoid groove is present, and the stapedial and sphenofrontal foramina are present, configuring the primitive stapedial and carotid circulatory pattern (pattern I). The suspensory process of squamosal is robust, overlapped to a small and wide tegmen tympanii, configuring a small postglenoid foramen; the subsquamosal fenestra is absent. The auditory meatus is wide; the mastoid is rounded and large, perforated by a large fenestra. The incisive foramina are short and moderately wide (Table 3), with anterior and posterior margins rounded and lateral margins wider more anteriorly (lateral margins more convex); the posterior margin is distant from the molar series. The inferior root of zygomatic plate is well anterior to the upper molar series. The palate is very long and wide (Table 3), with simple and small posterolateral palatal pits. The mesopterygoid fossa is narrow, with anterior margin presenting a wide medial process; the anterior margin is distant from the molar series, the roof of mesopterygoid fossa is completely ossified, without vacuities. The parapterygoid fossae are narrow, slightly narrower than the mesopterygoid fossa; the fossae are perforated by small (and double) to moderate posterior opening of the alisphenoid canal. The foramen lacerum medium is very narrow, as the auditory bulla exhibits a very reduced stapedial process and is positioned far from the parapterygoid fossa. The wide bulla is flask shaped, with Eustachian tube wide and short; the auditory meatus penetrates on the ventral surface of the auditory bullae; the orbicular apophysis of malleus is quite reduced.

The mandible ramus is moderately low. The condyloid process is developed and the coronoid process is strongly developed, falciform, and much higher than the condyloid process; the angular process is short, levelled to the condyloid process; the inferior and superior notches are shallow. The capsular process of the lower incisor is inconspicouos. The retromolar fossa is narrow and deep.

**Figure 7:**
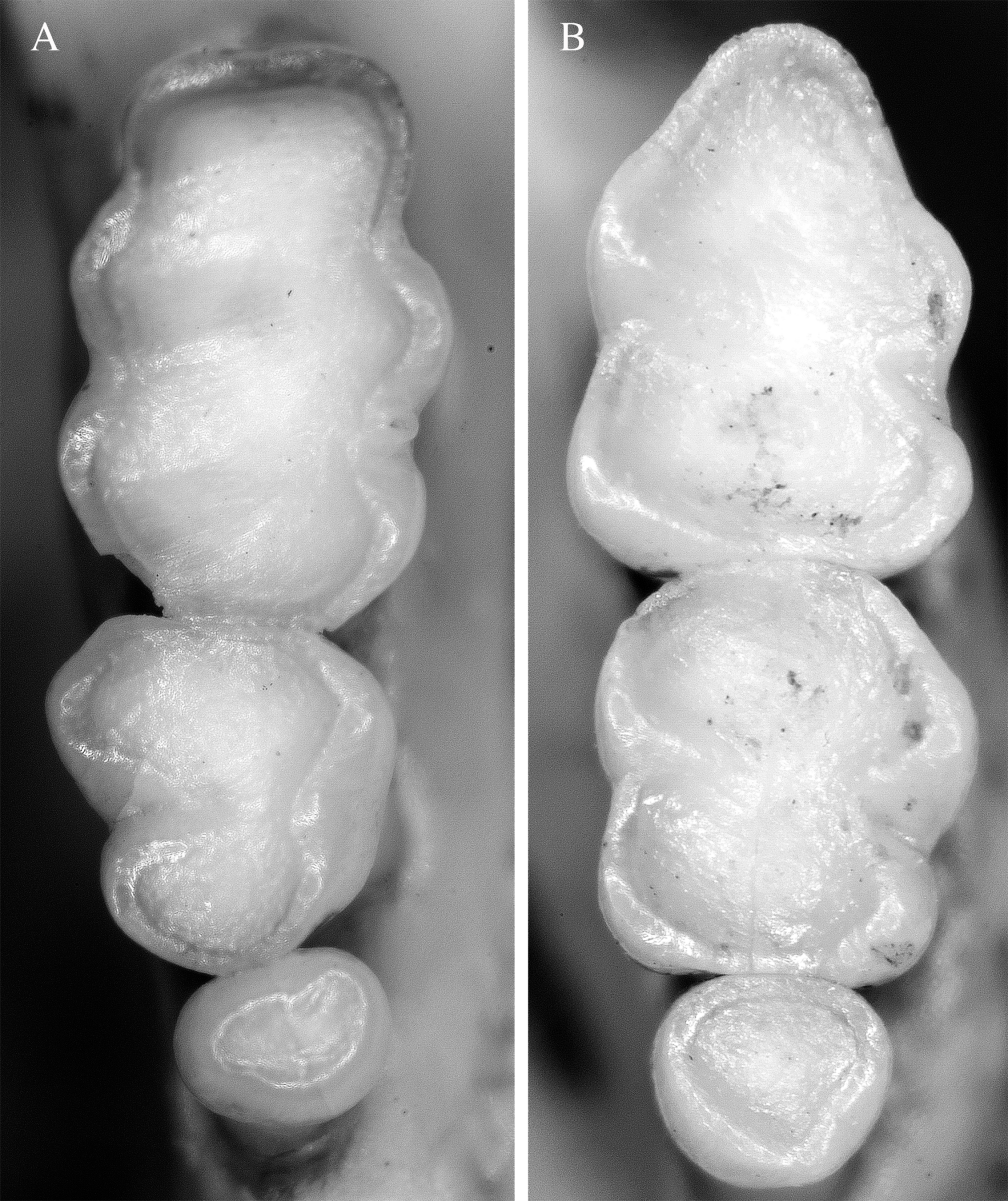
Molar series of *Neusticomys peruviensis*. Plate with molar series of the *Neusticomys peruviensis*: Right upper series (A); right lower series (B); both from specimen MTR25579, from Pacaás Novos, Rondônia.

Upper incisors are very robust, wide and deep; opisthodont. Upper molars series are small and delicate, well-worn (class 4); series are slightly convergent posteriorly; main cusps are arranged in slightly alternate pairs, the lingual positioned posteriorly than the labial ones; flexus are not very conspicuous on the molar topography. The M1 (Fig. 7) presents a wide procingulum, with a very shallow anterior notch restricted to the enamel surface. The anteroloph is not visible. The paracone and protocone are connected posteromedially to the median mure. The mesoloph is not visible, probably fused to the paracone. The metacone-hypocone pair is subequal to paracone-protocone pair. The posteroloph is present, but fused to metacone; posteroloph short, not reach the molar surface. The M2 is quite similar to M1; the metacone-hypocone pair is narrower than paracone-protocone pair; posteroloph not visible. The M3 is very small, peg-like. Lower molars also with cusps arranged in alternate pairs, the lingual anteriorly positioned to the labial. The m1 exhibits a very small and narrow procingulum, medially positioned and without conspicuous flexid. The metaconid-paraconid pair is subequal to the entoconid-hypoconid pair. The mesolophid and the posteroloph are not visible. The m2 is very similar to m1; the metaconid-paraconid pair is much narrower than the entoconid-hypoconid pair. The m3 is also peg-like.

The morphological variation of MRT25579 fits the morphology of *Neusticomys peruviensis* (Musser & Gardner, 1974), as reviewed by Voss (1988, 2015) and Percequillo, Carmignotto & Silva (2005). Our specimen and the type specimen share a more orange-copper dorsal and lateral coloration, as well as ears and hindfeet with brown-orange hairs and long and dense mystacial vibrissae. They also share a more robust skull, with inferior zygomatic root positioned well anterior to the upper molar series, and very small orbicular apophysis. The specimen from Rondônia is slightly smaller than the holotype of *N. peruviensis* in few cranial traits (Table 3), more noticeably on the length of the upper molar series, a usual diagnostic trait for this species, and also on the length of incisive foramina.

**Figure 8:**
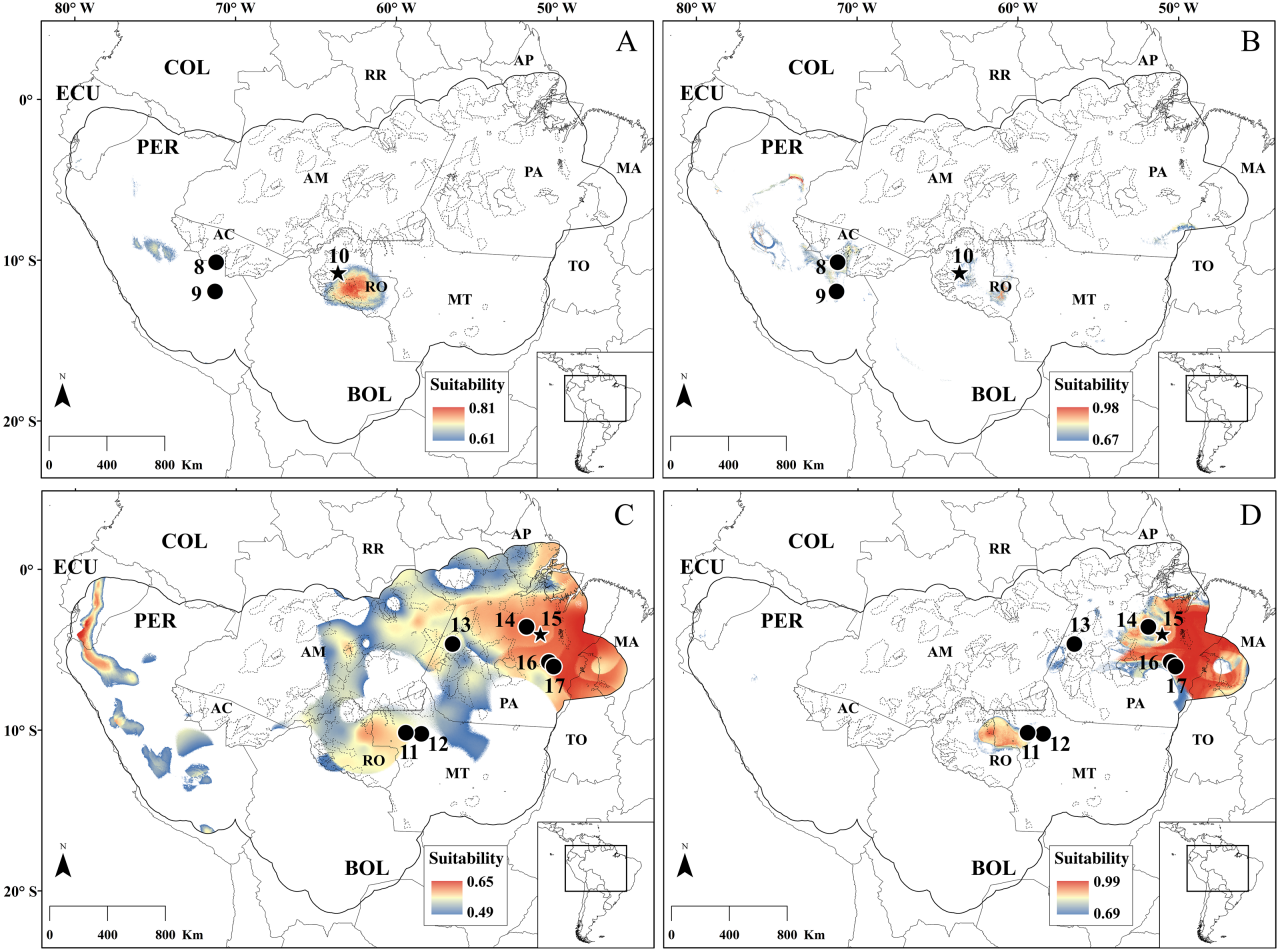
SDM for the species of genus *Neusticomys*. Species distribution models for *N. peruviensis* (A–B) and for *N. ferreirai* (C–D) built with MaxEnt (A–C) and RF (B–D). Dots represent the points used in building the model, and the stars are the new occurrence points reported in this study. Continuous gray line represent the political limits, continuous black line represent the modeled area (see text for explanation), and the dotted black line represent the limits of Brazilian conservation units (on federal, state and municipal levels). Minimum and maximum suitability values are represented by different colors. Localities numbers are listed in Table 2. For acronyms, see legend of Fig. 4.

The two known records for *N. peruviensis* (Table 2), along with the new record presented here, were employed on the SDM analysis. The most important variable for MaxEnt model was Temperature Seasonality (BIO04: 0.38), and for RF model was Potential Evapo-Transpiration in January (PET01: 0.71) (see Data S2). Likewise, as in the *R. longilingua* models, the AUC showed good performance for both algorithm (MaxEnt: 0.851; RF: 0.997) while TSS well performed only for RF (MaxEnt: 0.023; RF: 0.993). The main areas of occurrence indicated by the models are in the Western Amazon, in areas near the Andes foothills, and disjunct areas in the south of the state of Pará for RF, and in the south and center of the state of Rondônia (Fig. 8). Unlike *R. longilingua* models, suitable areas recovered by MaxEnt were similar in size than RF model (MaxEnt: 112,078.69 km^2^; RF: 80,255.04 km^2^). However, MaxEnt did not predict *N. peruviensis* near the known localities in Peru while the RF predicted suitable area near to all known occurrence points.

The specimen from Pacajá, Pará, X1M27, is a young female with erupting M3 (age class 2/o/i), captured on January 26, 2015, through pitfall traps placed on a flat lowland “terra firme” forest interspersed by several small streams, at an elevation of 223 m. X1M27 is prepared as skin and cleaned skull, and hepatic tissue preserved in ethanol (98%), and stored at −20 °C. The morphology of this individual (Table 3, Fig. 6) is quite similar to that described in literature for the genus (see Voss, 1988; Percequillo, Carmignotto & Silva, 2005), except for some traits that will be detailed thereafter. As a young individual, this individual presents a dorsal body color grayish-brown scarcely and finely grizzled with ochre; the lateral portion of the body is predominantly grayish; the ventral region is also grayish, but lighter than lateral region, and the transition from lateral to ventral portion is subtle. The head is similar to the dorsal pelage; ears are small and rounded (Table 3), almost concealed by the fur, densely covered by moderately long hairs on the internal surface and on the external margin; internal hairs are entirely brown. Fore and hind feet small (Table 3), densely covered by short brown hairs; the ungual tufts are very sparse and short; interdigital pads and thenar pad are fleshy, hypothenar pad not visible, apparently absent. The tail is quite shorter than head and body (Table 3), densely hirsute, with a short apical tuft (hairs ranging from 6 to 7 mm in length); the tail is unicolored, brownish, with brownish apical hairs. The mystacial vibrissae are less dense and moderately long (ranging from 26 to 29 mm, in length), slightly surpassing ears when laid backwards. The skull is small and delicate; the interorbital region is hourglass-shaped, with supraorbital margins rounded; temporal margins absent. The incisive foramina are moderately long and wide, with anterior and posterior margins rounded and lateral margins wider medially; the posterior margin is close to the molar series. The inferior root of zygomatic plate is slightly anterior to the upper molar series. The orbicular apophysis of malleus is absent. Upper incisors are delicate; orthodont. Regarding the molars, in M2 the metacone-hypocone pair is quite narrower than paracone-protocone pair.

The traits above presented for the specimen X1M27 fits the description of *Neusticomys ferreirai* Percequillo, Carmignotto & Silva (2005), and we, therefore, recognize this as a new record for this species in northeastern Pará. Quantitatively, data from the specimen from Pacajá is also more similar to the smaller species of the genus, more precisely to *N. ferreirai*, rather than to *N. peruviensis*. As a young individual, some differences from the nominotypical specimens are also visible (Table 3), but in few variables and consisting of smaller size.

For *N. ferreirai*, there are six known localities in the states of Mato Grosso and Pará, plus the new locality furnished here (Table 2). Models built with these seven occurrence points had the same patterns in performance metrics; good results for AUC in both algorithms (MaxEnt: 0.876; RF: 0.974) and for TSS only RF had satisfactory performance (MaxEnt: 0.489; RF: 0.987). In both algorithms, the most important variable was Temperature Seasonality BIO04 (MaxEnt: 0.64; RF: 0.57). The RF model indicated areas close to the points of occurrence already known as western Pará, northern Mato Grosso and east of Pará as the ones with the highest probability of occurrence for this species. There are only a few pixels with high probability of occurrence in the Andean region of Peru and Ecuador. In turn, MaxEnt model presenting larger suitable areas in modelled region, a pattern similar to *R. longilingua* (MaxEnt: 1,999,348.45 km^2^; RF: 483,936.13 km^2^). Both algorithms recovered suitable areas near to previously known collection points. For *N. peruviensis,* the models predicted potential occurrence areas in the central Peru, and in the states of Rondônia and Pará in Brazil. The areas in Rondônia showed the highest suitability values. For *N. ferreirai*, we revealed large highly suitable areas in Peru and in the eastern Amazon basin, again highlighting the State of Pará.

## Discussion

### Sampling

All specimens recorded here were sampled with pitfall traps. Two of the three recently specimens of *R. longilingua* reported by  Medina et al. (2017) were captured with pitfall traps, as well as the recently reported specimens of genus *Neusticomys* (Percequillo, Carmignotto & Silva, 2005; Leite, Silva & Da Gardner, 2007; Miranda et al., 2012; Braga & Duda, 2017). Therefore, we emphasize that surveys using pitfall traps are essential for the capture of these rodents, once again reinforcing the importance of this methodology on the study of Neotropical rodents (see Voss & Emmons, 1996; Abreu-Júnior et al., 2016; Abreu-Júnior, Brennand & Percequillo, 2017).

### Morphology and species definition

Comparing the description above provided for *R. longilingua* with the original description (Luna & Patterson, 2003), it is clear that most external and cranial diagnostic traits described for the species are observable in this new specimen. However, this specimen presents three hair types instead of two, as stated by Luna & Patterson (2003). Moreover, we believe that the setiforms are modified into spines rather than the aristiforms (as suggested by Luna & Patterson, 2003), which are uncommon and restricted to the rump region, exceeding in length the spiny fur. On the molar series, MJ550 lacks the anterolingual conule and anterocrista on M1, as well as the entoconid and the posterocrista on m3; on the m2, there is a small oblique projection posterior to the hypoconid, which could be assigned as a posterocrista, but as the tooth exhibits little wear it is difficult to confidently identify the structure.  Medina et al. (2017) reported that all three specimens recently obtained by them from Peru, exhibit the anterolingual conule and the posterocrista. Nevertheless, the absence of these structures in this new specimen suggests that they are polymorphic for the species, not configuring an exclusive diagnostic feature of *R. longilingua* as originally proposed (Luna & Patterson, 2003). Therefore, a polymorphism regarding the presence or absence of the posterocrista renders difficult the identification of the individual of *Rhagomys* recently reported for Alta Floresta, Mato Grosso (Percequillo et al., 2011). Both Peruvian and Brazilian specimens of *R. longilingua* exhibit reduced/absent anterolingual conules, whereas specimens of *R. rufescens* show these conules well developed; the specimen from Alto Floresta shares with *R. longilingua* this reduction on the procingulum of M1.

The specimens of *N. peruviensis* (MTR25579) and *N. ferreirai* (X1M27) here presented are quite similar to the original descriptions of these two species, with some minor differences that we interpret as individual or geographic variation. Nevertheless, as more specimens become available, more variation within and among species is being described and more overlap is being acknowledged between qualitative and quantitative traits. So far, this new evidence does not invalidate their specific status, but in the near future a re-evaluation will be mandatory.

### Collection localities and SDM

*Rhagomys* and *Neusticomys* are among the rarest sigmodontine rodents in South America, being known ffrom few specimens obtained in a handful of localities (see Voss, 1988; Luna & Patterson, 2003; Percequillo, Gonçalves & Oliveira, 2004; Percequillo, Carmignotto & Silva, 2005; Voss, 2015) *Rhagomys longilingua* have been collected in six previous known localities, one in moderate elevation of lowland Amazonian Forest, in Maskoitania, Peru (Fig. 4; Table 2; locality 4), and five in elevations above 900 m in Peru and Bolivia. The present record of *R. longilingua* is from an elevation of 105.1 m in Amazonian lowlands and extends the distribution of the collection localities of the species about 700 km eastwards.  Medina et al. (2017) also recovered this species ranging from 936 to 2,200 m of elevation on the additional localities they provide in Peru, stating that current elevational distribution lies between 450 and 2,200 m, but our records show that *R. longilingua* presents a wider elevation amplitude. Topographic variables showed to be important in explaining the species distribution of *R. longilingua*, even using the most common climatic variables in SDM studies (WorldClim variables; Hijmans et al., 2005). These results corroborates results of Wilson et al. (2013) who recommend the incorporation of remote sensing variables into SDM to improve model reliability and the statistical indexes. The model generated by the RF, despite having better statistical indexes than the MaxEnt, seems much attached to the topography and it did not predict the occurrence of the species in lowland Amazonian Forest. For this reason, even with low TSS, the MaxEnt model for this species seems to be more biologically coherent. There are one additional record for *Rhagomys* in Central Brazilian Amazonia without specific identification (Percequillo et al., 2011). There are suitable areas around this record (09°53′S, 56°28′W, 284 m) in our MaxEnt model for the occurrence of *R. longilingua*. Additionally, we narrowed the distance from this record of Alta Floresta to nearest *R. longilingua* record (our new record from UHE Jirau) to approximately 900 km. Considering the “close” distribution, the similarity on some dental traits (small/reduced anterolingual conule on M1) it is likely that the specimen from Mato Grosso could represent an individual of *R. longilingua*. Nevertheless, it is still tentative to confidently associate this specimen to *R. longilingua*, considering the morphological variation of this species.

The six species of the ichthyomyine *Neusticomys* are distributed over 28 localities, being *N. oyapocki*, from northern Amazonia, and *N. monticolus*, from Colombian and Ecuadorean Andes, the species with most known collection localities (Hanson et al., 2015; Voss, 2015). The new sample of *N. ferreirai* represents the seventh known locality (Table 2) but it does not extend the geographical distribution of this species. Analogously to the models recovered for *Rhagomys longilingua*, the MaxEnt model for *N. ferreirai* was broader and with smaller TSS value than the RF model. The most important variable for this species, in both algorithms, was the seasonality of temperature. This variable indicates consistency between the models and the natural history of the species, because *N. ferreirai* is a small semiaquatic rodent, and there is little temperature variation in this habitat when compared to terrestrial ecosystems (Langan et al., 2001; Dalapicolla, 2014). Both models for *N. ferreirai* are biologically coherent but RF model is more robust in statistical indexes. More distribution data are needed to evaluate which of the models, the broader (MaxEnt) or the restricted one (RF), best represent the suitable areas for *N. ferreirai*.

Regarding *N. peruviensis*, this is the third known specimen of the species, and allowed us to extend the distribution of this species approximately 650 km eastwards. As in previous results, the RF model exhibited robust statistical support. However, in this case, RF model has more biological coherence than the MaxEnt model, which did not recover suitable areas near to the points of occurrence in Peru. In both models, climatic variables were the most important, namely seasonality of the temperature (*N. peruviensis* is also semiaquatic) and potential evapotranspiration. Potential evapotranspiration is dependent of the vegetation cover and high rates of evapotranspiration are characteristic of forested areas (Camargo & Camargo, 2000), aspects that are consistent with the forest habitat of this species. It is important to note that for the two species of *Neusticomys*, areas with high environmental suitability were recovered in the same regions of Peru, Rondônia, and southeastern Pará. This would suggest: (i) the possibility of sympatry among these species; (ii) that their environmental requirements are similar; or (iii) that there is only one species throughout the Southern Amazonia, from eastern Peru to eastern Pará, Brazil. These assumptions further indicate, as our morphological results, that a revision of their taxonomic status is needed as new samples become available in the future.

The objective of this work was not to compare the algorithms but to identify potential areas for new records of these rare rodent species. However, we showed that MaxEnt presented suitable areas broader than the RF for two species, and in the third one the recovered area was similar in the two models. Broader areas are expected in MaxEnt models because minimum presence threshold (LTP) was used for the construction of these models (Radosavljevic & Anderson, 2014), with minimum values of suitability between 0.1 and 0.6 (Figs. 4 and 8). For the RF models only models with TSS and AUC above 0.7 were used in the average model, which generated minimum values of suitability around 0.7 (Figs. 4 and 8). If we analyzed only the most suitable areas, they are very similar, while the discordant areas between algorithms were those that exhibit low suitability. In other words, the MaxEnt model cut by the LTP presents greater commission error (areas with low suitability), an interesting result in studies like ours, that aim to recognize new areas of occurrence.

RF models had better performing metrics than MaxEnt models, but this was not reflected in more coherent models with species natural history, thus the MaxEnt models seemed more appropriated. MaxEnt was often used for modelling rare species (Parolo, Rossi & Ferrarini, 2008; Gogol-Prokurat, 2011; Bean, Stafford & Brashares, 2012; Gutiérrez et al., 2015). However, in the last years new algorithms like RF have gained space presenting more robust statistical results for species with small sample size (Cutler et al., 2007; Evans et al., 2011; Mi et al., 2017). The choice between different algorithms and methods, in many studies are done performing metrics of comparisons, such as AUC and TSS (Elith & Graham, 2009; Jiménez-Valverde, 2012; Breiner et al., 2015). The biological interpretation of the results is rarely taken into account, and the results presented here show that some models, even if not showing the best performance metrics, can explain more appropriately the distribution of the species, especially with small sample size. This increases the importance of the researcher’s knowledge on the natural history of the target species for a more biological and, possibly, better interpretation of the models.

In addition to the difficulties of modeling species with small sample size, this study presented another challenge, i.e., modeling in the Amazon region. Few studies with SDM were made in the Amazon (Hopkins, 2007; Saatchi et al., 2008; Cayuela et al., 2009), especially the Western Amazon, when compared with other biomes in South America as the Atlantic Forest (Tonini, Costa & Carnaval, 2013; Carnaval et al., 2014; Leite et al., 2016). A way to improve the Amazonian SDM is the use of remote sensing predictors, in addition to the variables constructed by interpolation (Bradley & Fleishman, 2008; Wilson et al., 2013), and the control of the models complexity (Merow et al., 2014; Radosavljevic & Anderson, 2014).

Numerous methods to build SDM for species with few occurrence points are available (Pearson et al., 2007; Wisz et al., 2008; Lomba et al., 2010; Bean, Stafford & Brashares, 2012; Breiner et al., 2015). Most of them agree that evaluating the complexity of the models (by information criteria, specifically AICc, for example) increases the ecological plausibility of the results (Galante et al., 2017). However, we agree that the power of models to predict areas where these species occur is smaller than in models build with small sample size. Wisz et al. (2008) suggested that models made with few points of occurrence should not be used in complex applications. However, they suggested these SDM can be used to prioritize survey area to collect rare species and to infer missing information on species ecology, as geographical distribution. In fact, in the literature, there are several examples of studies that use SDM to optimize collections of rare species (Guisan et al., 2006) or to find new populations of endemic habitat species (Rhoden, Peterman & Taylor, 2017). Thus, we believe that even we cannot use these SDM to do deeper conclusions on ecological niches they are important to indicate areas for future sampling and new information on their geographic distribution.

Regarding variation on elevational distribution, our results showed that performance metrics of models (AUC and TSS) of lowland and mountain regions have similar values when we compare the same algorithm. Thus, we could not affirm there is evidence, based on our dataset and algorithms employed, that allow us to discuss the performance of the SDM methods in different elevations. Perhaps the approach to study this case would be to evaluate with SDM’s widely distributed species, both throughout the geography and elevational range. Then afterwards modelling, to compare these areas through external validation or survey in the field. Another well-discussed point regarding the SDM of species occurring mountainous and lowland areas is the relevance of environmental variables representing the elevation, topography, and slope of the terrain. Some studies have empirically demonstrated the presence of these variables may improve the quality of SDMs for high-elevation species (Wilson et al., 2013;  Oke &Thompson, 2015).  Medina et al. (2017) not listed the variables used in their *R. longilingua* models, which may explain the differences between our models (besides our new record of occurrence for Brazil). In our case, we did not perform models with and without variables related to elevation (since it was not an objective of our study, as our samples are small), and we are not able to measure the effect of these variables in the models. However, since one these variables were retained and contributed most for the modelling of *R. longilingua*, after the removal of multicollinearity, we can hypothesize that elevational variables are important for the explanation of the distribution of the species that occur in mountains, while climatic variables were retained to explain the distribution of *Neusticomys* species occurring in lowland Amazon.

### Distribution and conservation

It is noteworthy that the highest suitable areas for all these species in Brazil are coincident with the arc of deforestation (“arco do desmatamento”), the region with the highest deforestation rates along the Amazon biome (Fearnside, 2017; http://e360.yale.edu/features/business-as-usual-a-resurgence-of-deforestation-in-the-brazilian-amazon) and characterized by the presence of huge hydroelectric dams. In Brazil, the government is planning to construct at least 79 hydroelectric in Amazon, flooding about ten million of hectares (Fearnside, 2015). The new record of *R. longilingua* (site 7, Table 2) and the record of Senador Porfírio for *N. ferreirai* (site 14, Table 2) are both located in hydroelectric dam sites in Amazon biome, which means these localities will vanish. This region also presents a lower density of protected areas comparing to the central Amazon (see Figs. 4 and 8; Ministério do Meio Ambiente, 2017). Therefore, it is imperative for the better knowledge and preservation of these rare species in Brazil sampling and conservation efforts focused on the arc of deforestation.

Potential changes on the laws determining the need of environmental impact reports prior to the beginning of commercial enterprises (e.g., construction of roads, power plants, hydroelectric dams, large factories, pasture, crop plantations) under appreciation of the Brazilian Congress (PL 3729/04; http://www.camara.gov.br/proposicoesWeb/fichadetramitacao?idProposicao=257161) may cause two negative impacts on our diversity: (i) without control, there will be accelerated devastation of natural resources in Brazil, with consequent loss of diversity; and (ii) long term diversity inventory programs will also be reduced or cancelled, precluding an opportunity to sample faunas that would be eventually lost by the implementation of such enterprises. Our records of *R. longilingua* and *N. ferreirai*, as well as recent records of specimens of *N. ferreirai* (Leite, Da Silva & Gardner, 2007; Braga & Duda, 2017), are obtained during inventories or monitoring studies associated environmental impact studied demanded by governmental agencies. In recent years, less was done to enforce environmental protection agencies and actions have been taken (and others will follow) to relax the legislation to protect the environment under the discussion that increase agricultural production and energy matrix would be the engine for economic advancement (Drummond & Barros-Platiau, 2006; Loyola, 2014; Benchimol & Peres, 2015; Fearnside, 2015; Fearnside, 2016).

### How many species of mammals are there in Brazil?

Even though such number is quite important for several issues, from basic general information to conservation policies, exact figures are and always be tentative, depending upon several methodological, conceptual and personal perceptions. Currently, according to data assembled in the Taxonomic Catalogue of the Fauna of Brazil (http://fauna.jbrj.gov.br/fauna/listaBrasil/ConsultaPublicaUC/ConsultaPublicaUC.do), there are 720 species (and 773 valid nominal taxa) in Brazil. In this contribution, we added two species to this list, making 722 species (or 775 valid nominal taxa) of mammals in Brazil.

Brazil is home to 109 species of threatened mammals (around 15% of the country’s total richness and 3% of global richness; ICMBio, 2014; WorldBank, 2017). This number can double and many species can be categorized in higher threat categories when geospatial data are incorporated to assess ecological preferences and current availability of habitats (Ocampo-Peñuela et al., 2016). Since the first richness list accounting 245 species published in 1648, ‘*Historia Naturalis Brasilae*’ by George Marcgrave (Vanzolini, 1996), the faunal richness of Brazil has not stopped growing, making the country first on the list of the most diverse countries in the world (Mittermeier, Robles & Mittermeier, 1997; Costa et al., 2005).

Nevertheless, when published data inform that there are 722 species of mammals in Brazil, this is not only a number: it represents a synthesis of a consistent knowledge constructed by several personnel and institutions and also presents a stimulus and challenge to future academics to continue to assess Brazilian mammal diversity.

## Conclusions

Short term and long term inventories here conducted were successful in recording these rare species. Is it likely that a large amount of uncertainty of our results and discussions was a direct consequence of the small sample size available for these taxa. Nevertheless, the individuals sampled were assigned to current known species, *R. longilingua*, *N. peruviensis* and *N. ferreirai*, based on morphologic traits, despite some variation on some qualitative and quantitative traits, as the pelage coloration and molar topography. With forthcoming specimens, species assignment based on detailed quantitative analysis or molecular approaches must be employed to test our hypothesis. We extended the known current localities of all three species, and also hypothesized on the potential areas of occurrence that should be surveyed in forthcoming field surveys, to test the predictability power of our models and (if these models are adequate) to obtain additional individuals to refine their limits, both on morphology and distribution. I would remove this period, as the same idea is presented on the last phrase of the next paragraph.

Therefore, we added two rare and poorly known species to the already diverse assemblage of mammals in Brazil, numbering 722 species (or 775 valid nominal taxa). Although, today we have more information available than in 1996, when Vivo raised the question that opens this contribution, allowing more precise estimates on this diversity, it is imperative that mammalogists kept inventories and revisionary programs in progress to maintain this highly important and valuable information updated.

##  Supplemental Information

10.7717/peerj.4071/supp-1 Data S1 List of specimens examined Specimens examined of genera *Neusticomys* and *Rhagomys*.

10.7717/peerj.4071/supp-2 Data S2 Climatic variables employed in SDM List of 46 environmental variables used in the construction of species distribution models (SDM), derived from interpolation and remote sensing methods, with indications of their sources.

10.7717/peerj.4071/supp-3 Data S3 Environmental variables for SDM List of environmental variables selected for the construction of the models to each of the three species and the importance of these variables, and for Random Forest (RF) and MaxEnt. Variable importance is represented by the mean of all model replicates, and the summed values can be different from 1. See Material and Methods section for details of variables selection.

10.7717/peerj.4071/supp-4 Data S4 Raw data on external and cranial measurements for genera *Rhagomys* and *Neusticomys* External and cranial measurements of specimens examined of the genera *Rhagomys* and *Neusticomys*. Data for *N. mussoi* and *D. venezuelae* are from literature: Ochoa & Soriano (1991) and Voss (1988), Ochoa & Soriano (1991) and Voss, Lunde & Simmons (2001), respectively. Data for *R. longilingua* is from literature: Luna & Patterson (2003).

## Additional Information and Declarations

### Data Availability

The following information was supplied regarding data availability: The raw data is provided in the text and Supplemental Files.

## References

[ref-1] Abreu-Júnior EF, Brennand P, Percequillo AR (2017). Diversidade de Mamíferos do Baixo Rio Jufari, Roraima, Brasil. Papéis Avulsos de Zoologia.

[ref-2] Abreu-Júnior EF, Freitas MA, Lapenta MJ, Venancio NM, Franca DPF, Percequillo AR (2016). Marsupials and rodents (Didelphimorphia and Rodentia) of Upper Rio Acre, with new data on *Oxymycterus inca* Thomas, 1900 from Brazil. Check List.

[ref-3] Barbet-Massin M, Jiguet F, Albert CH, Thuiller W (2012). Selecting pseudo-absences for species distribution models: how, where and how many?. Methods in Ecology and Evolution.

[ref-4] Bean WT, Stafford R, Brashares JS (2012). The effects of small sample size and sample bias on threshold selection and accuracy assessment of species distribution models. Ecography.

[ref-5] Benchimol M, Peres C (2015). Widespread forest vertebrate extinctions induced by a mega hydroelectric dam in lowland Amazonia. PLOS ONE.

[ref-6] Bonvicino CR, Casado F, Weksler M (2014). A new species of *Cerradomys* (Mammalia: Rodentia: Cricetidae) from Central Brazil, with remarks on the taxonomy of the genus. Zoologia.

[ref-7] Bovendorp RS, Villar N, Abreu-Júnior EF, Bello C, Regolin AL, Percequillo AR, Galetti M (2017). Atlantic small-mammal: a dataset of communities of rodents and marsupials of the Atlantic Forests of South America. Ecology.

[ref-8] Bradley BA, Fleishman E (2008). Can remote sensing of land cover improve species distribution modelling?. Journal of Biogeography.

[ref-9] Braga C, Duda R (2017). New records and phylogenetic position of *Neusticomys ferreirai* (Rodentia: Cricetidae) Percequillo, Carmignotto and Silva, 2005 from the Amazon basin, northern Brazil. Mammalia.

[ref-10] Breiner FT, Guisan A, Bergamini A, Nobis MP (2015). Overcoming limitations of modelling rare species by using ensembles of small models. Methods in Ecology and Evolution.

[ref-11] Camargo AP de, Camargo MBP de (2000). Uma revisão analítica da evapotranspiração potencial. Bragantia.

[ref-12] Carleton MD (1980). Phylogenetic relationships in neotomine-peromyscine rodents (Muroidea) and a reappraisal of the dichotomy within New World Cricetinae. Miscellaneous Publications, Museum of Zoology, University of Michigan.

[ref-13] Carnaval AC, Waltari E, Rodrigues MT, Rosauer D, VanDerWal J, Damasceno R, Prates I, Strangas M, Spanos Z, Rivera D, Pie MR, Firkowski CR, Bornschein MR, Ribeiro LF, Moritz C (2014). Prediction of phylogeographic endemism in an environmentally complex biome. Proceedings of the Royal Society B: Biological Sciences.

[ref-14] Cayuela L, Golicher DJ, Newton AC, Kolb M, Alburquerque FS, Arets EJMM, Alkemade JRM, Pérez AM (2009). Species distribution modeling in the tropics: problems, potentialities, and the role of biological data for effective species conservation. Tropical Conservation Science.

[ref-15] Christoff AU, Vieira EM, Oliveira LR, Gonçalves JW, Valiati VH, Tomasi PS (2016). A new species of *Juliomys* (Rodentia, Cricetidae, Sigmodontinae) from the Atlantic Forest of Southern Brazil. Journal of Mammalogy.

[ref-16] Costa LP, Leite YLR, Mendes SL, Ditchfield AD (2005). Mammal Conservation in Brazil. Conservation Biology.

[ref-17] Cutler DR, Edwards TC, Beard KH, Cutler A, Hess KT, Gibson J, Lawler JJ (2007). Random forests for classification in ecology. Ecology.

[ref-18] Dalapicolla J (2014). Papel da hidrografia e do clima na estrutura genética do roedor semiaquático *Nectomys squamipes*.

[ref-19] D’Elía G, Pardiñas UFJ, Patton JL, Pardiñas UFJ, D’Elía G (2015). Subfamily Sigmodontinae Wagner, 1843. Mammals of South America.

[ref-20] Dormann CF, Elith J, Bacher S, Buchmann C, Carl G, Carré G, Marquéz JRG, Gruber B, Lafourcade B, Leitão PJ, Münkemüller T, McClean C, Osborne PE, Reineking B, Schröder B, Skidmore AK, Zurell D, Lautenbach S (2013). Collinearity: a review of methods to deal with it and a simulation study evaluating their performance. Ecography.

[ref-21] Drummond J, Barros-Platiau AN (2006). Brazilian Environmental Laws and Policies, 1934–2002: a critical overview. Law & Policy.

[ref-22] Elith J, Graham CH (2009). Do they? How do they? Why do they differ? On finding reasons for differing performances of species distribution models. Ecography.

[ref-23] Elith J, Leathwick JR (2009). Species distribution models: ecological explanation and prediction across space and time. Annual Review of Ecology, Evolution, and Systematics.

[ref-24] Evans JS, Murphy MA, Holden ZA, Cushman SA, Drew CA, Wiersma YF, Huettmann F (2011). Modeling species distribution and change using random forest. Predictive species and habitat modeling in landscape ecology.

[ref-25] Fearnside PM (2015). Hidrelétricas na Amazônia: impactos ambientais e sociais na tomada de decisões sobre grandes obras.

[ref-26] Fearnside PM (2016). Brazilian politics threaten environmental policies. Science.

[ref-27] Fearnside PM (2017). Business as usual: a resurgence of deforestation in the Brazilian Amazon. http://e360yale.edu/features/business-as-usual-a-resurgence-of-deforestation-in-the-brazilian-amazon.

[ref-28] Fonseca GAB, Herrmann G, Leite YLR, Mittermeier RA, Rylands AB, Patton JL (1996). Lista anotada dos mamíferos do Brasil. Occasional Papers in Conservation Biology.

[ref-29] Galante PJ, Alade B, Muscarella R, Jansa SA, Goodman SM, Anderson RP (2017). The challenge of modeling niches and distributions for data-poor species: a comprehensive approach to model complexity. Ecography.

[ref-30] Gardner AL (2008). Mammals of South America.

[ref-31] Gogol-Prokurat M (2011). Predicting habitat suitability for rare plants at local spatial scales using a species distribution model. Ecological Applications.

[ref-32] Guisan A, Broennimann O, Engler R, Vust M, Yoccoz NG, Lehmann A, Zimmermann NE (2006). Using niche-based models to improve the sampling of rare species. Conservation Biology.

[ref-33] Gutiérrez EE, Maldonado JE, Radosavljevic A, Molinari J, Patterson BD, Martínez CJM, Rutter AR, Hawkins MTR, Garcia FJ, Helgen KM (2015). The taxonomic status of *mazama bricenii* and the significance of the táchira depression for mammalian endemism in the Cordillera de Mérida, Venezuela. PLOS ONE.

[ref-34] Hanson JD, D’Elia G, Ayers SB, Cox SB, Burneo SF, Lee Jr TE (2015). A new species of fish-eating rat, genus *Neusticomys* (Sigmodontinae), from Ecuador. Zoological Studies.

[ref-35] Hijmans RJ, Cameron SE, Parra JL, Jones PG, Jarvis A (2005). Very high resolution interpolated climate surfaces for global land areas. International Journal of Climatology.

[ref-36] Hopkins MJG (2007). Modelling the known and unknown plant biodiversity of the Amazon Basin. Journal of Biogeography.

[ref-37] ICMBio (2014). Lista de Espécies Ameaçadas. http://www.icmbio.gov.br/portal/especies-ameacadas-destaque.

[ref-38] IUCN (2016). The IUCN Red List of Threatened species. http://www.iucnredlist.org/.

[ref-39] Jiménez-Valverde A (2012). Insights into the area under the receiver operating characteristic curve (AUC) as a discrimination measure in species distribution modelling. Global Ecology and Biogeography.

[ref-40] Langan SJ, Johnston L, Donaghy MJ, Youngson AF, Hay DW, Soulsby C (2001). Variation in river water temperatures in an upland stream over a 30-year period. Science of the Total Environment.

[ref-41] Leite RN, Da Silva MNF, Gardner T (2007). New records of *Neusticomys oyapocki* (Rodentia, Sigmodontinae) from a human-dominated forest landscape in northeastern Brazilian Amazonia. Mastozoologia Neotropical.

[ref-42] Leite YLR, Costa LP, Loss AC, Rocha RG, Batalha-Filho H, Bastos AC, Quaresma VS, Fagundes V, Paresque R, Passamani M, Pardini R (2016). Neotropical forest expansion during the last glacial period challenges refuge hypothesis. Proceedings of the National Academy of Sciences of the United States of America.

[ref-43] Liaw A, Wiener M (2002). Classification and regression by randomForest. R News.

[ref-44] Lomba A, Pellissier L, Randin C, Vicente J, Moreira F, Honrado J, Guisan A (2010). Overcoming the rare species modelling paradox: a novel hierarchical framework applied to an Iberian endemic plant. Biological Conservation.

[ref-45] Louzada NSV, Monte Lima AC, Pessôa LM, Cordeiro JLP, Oliveira LFB (2015). New records of phyllostomid bats for the state of Mato Grosso and for the Cerrado of Midwestern Brazil (Mammalia: Chiroptera). Check List.

[ref-46] Loyola R (2014). Brazil cannot risk its environmental leadership. Diversity and Distributions.

[ref-47] Luna L, Patterson BD (2003). A remarkable new mouse (Muridae: Sigmodontinae) from Southeastern Peru: with comments on the affinities of *Rhagomys rufescens* (Thomas, 1886). Fieldiana Zoology.

[ref-48] Medina CE, Días DR, Pino K, Pari A, Zeballos H (2017). New locality records of *Rhagomys longilingua* Luna & Patterson, 2003 (Rodentia: Cricetidae) in Peru. Check List.

[ref-49] Merow C, Smith MJ, Edwards TC, Guisan A, McMahon SM, Normand S, Thuiller W, Wüest RO, Zimmermann NE, Elith J (2014). What do we gain from simplicity versus complexity in species distribution models?. Ecography.

[ref-50] Mi C, Huettmann F, Guo Y, Han X, Wen L (2017). Why choose Random Forest to predict rare species distribution with few samples in large undersampled areas? Three Asian crane species models provide supporting evidence. PeerJ.

[ref-51] Ministério do Meio Ambiente (2017). Download de dados geográficos. http://mapas.mma.gov.br/i3geo/datadownload.htm.

[ref-52] Miranda CL, Rossi RV, Semedo TBF, Flores TA (2012). New records and geographic distribution extension of *Neusticomys ferreirai* and *N. oyapocki* (Rodentia, Sigmodontinae). Mammalia.

[ref-53] Mittermeier RA, Robles GP, Mittermeier CG (1997). Megadiversity: earth’s biologically wealthiest nations.

[ref-54] Mora C, Tittensor DP, Adl S, Simpson AGB, Worm B (2011). How many species are there on Earth and in the Ocean?. PLOS Biology.

[ref-55] Moratelli R, Dias D (2015). A new species of nectar-feeding bat, genus *Lonchophylla*, from the Caatinga of Brazil (Chiroptera, Phyllostomidae). Zookeys.

[ref-56] Muscarella R, Galante PJ, Soley-Guardia M, Boria RA, Kass JM, Uriarte M, Anderson RP (2014). ENMeval: an R package for conducting spatially independent evaluations and estimating optimal model complexity for Maxent ecological niche models. Methods in Ecology and Evolution.

[ref-57] Musser GG, Carleton MD, Brothers EM, Gardner AL (1998). Systematic studies of oryzomyine rodents (Muridae, Sigmodontinae): diagnoses and distributions of species formerly assigned to *Oryzomys* “*capito*”. Bulletin of the American Museum of Natural History.

[ref-58] Musser GG, Gardner AL (1974). A new species of ichthyomyine *Daptomys* from Peru. American Museum Novitates.

[ref-59] Nunes A (2002). First record of *Neusticomys oyapocki* (Muridae: Sigmodontinae) from the Brazilian Amazon. Mammalia.

[ref-60] Ocampo-Peñuela N, Jenkins CN, Vijay V, Li BV, Pimm SL (2016). Incorporating explicit geospatial data shows more species at risk of extinction than the current Red List. Science Advances.

[ref-61] Ochoa JG, Soriano P (1991). A new species of water rat genus *Neusticomys* Anthony, from the Andes of Venezuela. Journal of Mammalogy.

[ref-62] Oke OA, Thompson KA (2015). Distribution models for mountain plant species: the value of elevation. Ecological Modelling.

[ref-63] Oliveira TG, Mazim FD, Vieira OQ, Barnett APA, Silva GN, Soares JBG, Santos JP, Silva VF, Araújo PA, Tchaika L, Miranda CL (2016). Nonvolant mammal megadiversity and conservation issues in a threatened Central Amazonian hotspot in Brazil. Tropical Conservation Science.

[ref-64] Pacheco V, Vivar E, Wilson DE, Sandoval A (1996). Annotated checklist of the non-flying mammals at Pakitza, Manu Reserve Zone, Manu National Park, Peru. Manu, the biodiversity of Southeastern Peru.

[ref-65] Paglia AP, Fonseca GAB, Rylands AB, Herrmann G, Aguiar LMS, Chiarello AG, Leite YLR, Costa LP, Siciliano S, Kierulff MCM, Mendes SL, Tavares VC, Mittermeier RA, Patton JL (2012). Lista Anotada dos Mamíferos do Brasil.

[ref-66] Pardiñas UFJ, Teta P, Salazar-Bravo J, Myers P, Galliari CA (2016). A new species of arboreal rat, genus *Oecomys* (Rodentia, Cricetidae) from Chaco. Journal of Mammalogy.

[ref-67] Parolo G, Rossi G, Ferrarini A (2008). Toward improved species niche modelling: *Arnica montana* in the Alps as a case study. Journal of Applied Ecology.

[ref-68] Patton JL, Pardiñas UFJ, D’Elía G (2015). Mammals of South America.

[ref-69] Pavan S (2015). A new species of *Monodelphis* (Didelphimorphia: Didelphidae) from the Brazilian Atlantic Forest. American Museum Novitates.

[ref-70] Pearson RG, Raxworthy CJ, Nakamura M, Townsend Peterson A (2007). Predicting species distributions from small numbers of occurrence records: a test case using cryptic geckos in Madagascar. Journal of Biogeography.

[ref-71] Percequillo AR, Braga CAC, Brandão MV, Abreu-Júnior EF, Gualda-Barros J, Lessa GM, Pires MRS, Hingst-Zaher E (2017). The genus *Abrawayaomys* Cunha and Cruz, 1979 (Rodentia: Cricetidae: Sigmodontinae): geographic variation and species definition. Journal of Mammalogy.

[ref-72] Percequillo AR, Carmignotto AP, Silva MJJ (2005). A new species of *Neusticomys* (Ichthyomyini, Sigmodontinae) from Central Brazilian Amazonia. Journal of Mammalogy.

[ref-73] Percequillo AR, Gonçalves PR, Oliveira JA (2004). The rediscovery of *Rhagomys rufescens* (Thomas, 1886), with a morphological rediscription and comments on its systematic relationships based on morphological and molecular (cytochrome b) characters. Mammalian Biology.

[ref-74] Percequillo AR, Gregorin R (2017). Catálogo Taxonômico da Fauna do Brasil. http://fauna.jbrj.gov.br/fauna/faunadobrasil/64.

[ref-75] Percequillo AR, Tirelli FP, Michalski F, Eizirik E (2011). The genus *Rhagomys* (Thomas 1917) (Rodentia, Cricetidae, Sigmodontinae) in South America: morphological considerations, geographic distribution and zoogeographic comments. Mammalia.

[ref-76] Phillips SJ, Anderson RP, Schapire RE (2006). Maximum entropy modeling of species geographic distributions. Ecological Modelling.

[ref-77] Pinheiro P, Hartmann P, Geise L (2004). New record of *Rhagomys rufescens* (Thomas 1886) (Rodentia: Muridae). Zootaxa.

[ref-78] R Core Team (2014). https://www.r-project.org.

[ref-79] Radosavljevic A, Anderson RP (2014). Making better Maxent models of species distributions: complexity, overfitting and evaluation. Journal of Biogeography.

[ref-80] Reig OA (1977). A proposed unified nomenclature for the enamelled components of the molar teeth of the Cricetidae (Rodentia). Journal of Zoology.

[ref-81] Reis NR, Peracchi AL, Pedro VA, Lima IP (2006). Mamíferos do Brasil.

[ref-82] Reis NR, Peracchi AL, Pedro VA, Lima IP (2011). Mamíferos do Brasil.

[ref-83] Rhoden CM, Peterman WE, Taylor CA (2017). Maxent-directed field surveys identify new populations of narrowly endemic habitat specialists. PeerJ.

[ref-84] Rossi RV, Miranda CL, Semedo TBF (2016). Rapid assessment of nonvolant mammals in seven sites in the northern State of Pará, Brazil: a forgotten part of the Guiana Region. Mammalia.

[ref-85] Saatchi S, Buermann W, Steege H, Mori S, Smith TB (2008). Modeling distribution of Amazonian tree species and diversity using remote sensing measurements. Remote Sensing of Environment.

[ref-86] Shcheglovitova M, Anderson RP (2013). Estimating optimal complexity for ecological niche models: a jackknife approach for species with small sample sizes. Ecological Modelling.

[ref-87] Sikes RS, Animal Care and Use Committee of the American Society of Mammalogists (2016). Guidelines of the American Society of Mammalogists for the use of wild mammals in research and education. Journal of Mammalogy.

[ref-88] Tabachnick BG, Fidell LS (2007). Using multivariate statistics.

[ref-89] Thuiller W, Georges D, Engler R, Breiner F (2016).

[ref-90] Tonini JFR, Costa LP, Carnaval AC (2013). Phylogeographic structure is strong in the Atlantic Forest; predictive power of correlative paleodistribution models, not always. Journal of Zoological Systematics and Evolutionary Research.

[ref-91] Vanzolini PE (1996). Brasil dos Viajantes. Revista USP.

[ref-92] Villalpando G, Vargas J, Salazar-Bravo J (2006). First record of *Rhagomys* (Mammalia: Sigmodontinae) in Bolivia. Mastozoologia Neotropical.

[ref-93] Vivo M, Bicudo CEM, Menezes NA (1996). How many species of mammals there are in Brazil?. Biodiversity in Brazil. A first approach.

[ref-94] Voss RS (1988). Systematics and ecology of Ichthyomyine rodents (Muroidea): patterns of morphological evolution in a small adaptive radiation. Bulletin of the American Museum of Natural History.

[ref-95] Voss RS (1991). An introduction to the neotropical muroid rodent genus *Zygodontomys*. Bulletin of the American Museum of Natural History.

[ref-96] Voss RS, Patton JL, Pardiña UFJ, D’Elía G (2015). Tribe Ichthyomyini Vorontsov, 1959. Mammals of South America.

[ref-97] Voss RS, Emmons LH (1996). Mammalian diversity in neotropical lowland rainforests: a preliminary assessment. Bulletin of the American Museum of Natural History.

[ref-98] Voss RS, Lunde DP, Simmons NB (2001). The Mammals of Paracou, French Guiana: a neotropical lowland rainforest fauna Part 2. Nonvolant species. Bulletin of the American Museum of Natural History.

[ref-99] Wilson JW, Sexton JO, Todd Jobe R, Haddad NM (2013). The relative contribution of terrain, land cover, and vegetation structure indices to species distribution models. Biological Conservation.

[ref-100] Wisz MS, Hijmans RJ, Li J, Peterson AT, Graham CH, Guisan A (2008). Effects of sample size on the performance of species distribution models. Diversity and Distributions.

[ref-101] WorldBank (2017). Mammal species, threatened. http://data.worldbank.org/indicator/ENMAMTHRDNO.

