# Supplementary material for: How many species of mammals are there in Brazil? New records of rare rodents (Rodentia: Cricetidae: Sigmodontinae) from Amazonia raise the current known diversity
Source: PeerJ. 2017 Dec 15;5:e4071. doi: 10.7717/peerj.4071 (PMC5733914; doi:10.7717/peerj.4071)
Supplement: Data S1 — Specimens examined of genera Neusticomys and Rhagomys. [file peerj-05-4071-s001.docx]

**How many species of mammals are there in Brazil? New records of rare rodents (Rodentia: Cricetidae: Sigmodontinae) from Amazonia raise the current known diversity**

Alexandre R Percequillo, Jeronymo Dalapicolla, Edson F Abreu Júnior, Paulo Ricardo O Roth, Katia M P M B Ferraz, Elisandra A Chiquito

**Data S1**. Specimens Examined of genera *Neusticomys* and *Rhagomys*.

*Neusticomys ferreirai*: Brazil: Mato Grosso: Juruena: MZUSP 32092, 32093 (skin/skull); Pará: Pacajá: X1M27 (skin/skull).

*Neusticomys peruviensis*: Brazil: Rondônia: Parque Nacional de Pacaás Novos: MTR 25579 (skin/skull).

*Rhagomys longilingua*: Peru: Cuzco: Manu Biosphere Reserve: M: MUSM 17013 (skin), F: FMNH 170687 (skin/skull); Brazil: Rondônia: UHE Jirau: MJ 550 (skin/skull).

*Rhagomys rufescens*: U: MZUSP 31952; Brazil: Minas Gerais: Viçosa: Mata do Paraíso: MN 66056 (skin/skull); São Paulo: Estação Ecológica do Bananal: M: EEB 849 (skin/skull); Ribeirão Grande: F: AB 401 (fluid/skull); M: AB356 (fluid/skull); Rio de Janeiro: Rio de Janeiro: F: BMNH 86.2.8.5 (skin/skull).
