# Supplementary material for: How many species of mammals are there in Brazil? New records of rare rodents (Rodentia: Cricetidae: Sigmodontinae) from Amazonia raise the current known diversity
Source: PeerJ. 2017 Dec 15;5:e4071. doi: 10.7717/peerj.4071 (PMC5733914; doi:10.7717/peerj.4071)
Supplement: Data S2 — List of 46 environmental variables used in the construction of species distribution models (SDM), derived from interpolation and remote sensing methods, with indications of their sources. [file peerj-05-4071-s002.docx]

**How many species of mammals are there in Brazil? New records of rare rodents (Rodentia: Cricetidae: Sigmodontinae) from Amazonia raise the current known diversity**

Alexandre R Percequillo, Jeronymo Dalapicolla, Edson F Abreu Júnior, Paulo Ricardo O Roth, Katia M P M B Ferraz, Elisandra A Chiquito

**Data S2.** List of 46 environmental variables used in the construction of species distribution models (SDM), derived from interpolation and remote sensing methods, with indications of their sources.

| **Code** | **Description** | | **Method** | | **Type** | **Source** | |
| --- | --- | --- | --- | --- | --- | --- | --- |
| BIO01 | Annual Mean Temperature | | Interpolation | | Climatic | WorldClim | |
| BIO02 | Mean Diurnal Range (Mean of monthly (max temp - min temp)) | | Interpolation | | Climatic | WorldClim | |
| BIO03 | Isothermality (BIO2/BIO7) (* 100) | | Interpolation | | Climatic | WorldClim | |
| BIO04 | Temperature Seasonality (standard deviation *100) | | Interpolation | | Climatic | WorldClim | |
| BIO05 | Max Temperature of Warmest Month | | Interpolation | | Climatic | WorldClim | |
| BIO06 | Min Temperature of Coldest Month | | Interpolation | | Climatic | WorldClim | |
| BIO07 | Temperature Annual Range (BIO5-BIO6) | | Interpolation | | Climatic | WorldClim | |
| BIO08 | Mean Temperature of Wettest Quarter | | Interpolation | | Climatic | WorldClim | |
| BIO09 | Mean Temperature of Driest Quarter | | Interpolation | | Climatic | WorldClim | |
| BIO10 | Mean Temperature of Warmest Quarter | | Interpolation | | Climatic | WorldClim | |
| BIO11 | Mean Temperature of Coldest Quarter | | Interpolation | | Climatic | WorldClim | |
| BIO12 | Annual Precipitation | | Interpolation | | Climatic | WorldClim | |
| BIO13 | Precipitation of Wettest Month | | Interpolation | | Climatic | WorldClim | |
| BIO14 | Precipitation of Driest Month | | Interpolation | | Climatic | WorldClim | |
| BIO15 | Precipitation Seasonality (Coefficient of Variation) | | Interpolation | | Climatic | WorldClim | |
| BIO16 | Precipitation of Wettest Quarter | | Interpolation | | Climatic | WorldClim | |
| BIO17 | Precipitation of Driest Quarter | | Interpolation | | Climatic | WorldClim | |
| BIO18 | Precipitation of Warmest Quarter | | Interpolation | | Climatic | WorldClim | |
| BIO19 | Precipitation of Coldest Quarter | | Interpolation | | Climatic | WorldClim | |
| AI_YR | Global Aridity Index (Global-Aridity) | | Interpolation | | Land Cover | CGIAR-CSI | |
| PET_ANNUAL | Global Potential Evapo-Transpiration (Global-PET) - Annual | | Interpolation | | Land Cover | CGIAR-CSI | |
| PET01 | Global Potential Evapo-Transpiration (Global-PET) - January | | Interpolation | | Land Cover | CGIAR-CSI | |
| PET02 | Global Potential Evapo-Transpiration (Global-PET) - February | | Interpolation | | Land Cover | CGIAR-CSI | |
| PET03 | Global Potential Evapo-Transpiration (Global-PET) - March | | Interpolation | | Land Cover | CGIAR-CSI | |
| PET04 | Global Potential Evapo-Transpiration (Global-PET) - April | | Interpolation | | Land Cover | CGIAR-CSI | |
| PET05 | | Global Potential Evapo-Transpiration (Global-PET) - May | Interpolation | Land Cover | | | CGIAR-CSI |
| PET06 | | Global Potential Evapo-Transpiration (Global-PET) - June | Interpolation | Land Cover | | | CGIAR-CSI |
| PET07 | | Global Potential Evapo-Transpiration (Global-PET) - July | Interpolation | Land Cover | | | CGIAR-CSI |
| PET08 | | Global Potential Evapo-Transpiration (Global-PET) - August | Interpolation | Land Cover | | | CGIAR-CSI |
| PET09 | | Global Potential Evapo-Transpiration (Global-PET) - September | Interpolation | Land Cover | | | CGIAR-CSI |
| PET10 | | Global Potential Evapo-Transpiration (Global-PET) - Octuber | Interpolation | Land Cover | | | CGIAR-CSI |
| PET11 | | Global Potential Evapo-Transpiration (Global-PET) - November | Interpolation | Land Cover | | | CGIAR-CSI |
| PET12 | | Global Potential Evapo-Transpiration (Global-PET) - December | Interpolation | Land Cover | | | CGIAR-CSI |
| ETMNTS | | Long-Term MODIS-Estimated Evapotranspiration (MOD16) | Remote Sensing | Land Cover | | | WorldGrids |
| EVMMOD | | Mean Value of the Monthly MODIS EVI Time Series Data | Remote Sensing | Land Cover | | | WorldGrids |
| INMSRE | | Mean Potential Incoming Solar Radiation (8-Day Average) | Remote Sensing | Land Cover | | | WorldGrids |
| LAMMOD | | Mean Value of the 8-Day MODIS LAI Time Series Data | Remote Sensing | Land Cover | | | WorldGrids |
| MGVF | | Annual Maximum Green Vegetation Fraction (MGVF) | Remote Sensing | Land Cover | | | USGS |
| TREECOVER | | Tree Cover | Remote Sensing | Land Cover | | | GLCF |
| ALT | | Altitude | Remote Sensing | Topographic | | | WorldClim |
| OPISRE | | Topographic Openess Index | Remote Sensing | Topographic | | | WorldGrids |
| SLPSRT | | Slope in percent | Remote Sensing | Topographic | | | WorldGrids |
| TWISRE | | Topographic Wetness Index | Remote Sensing | Topographic | | | WorldGrids |
| ASP | | Aspect | Remote Sensing | Topographic | | | WorldGrids |
| SLOPE | | Slope | Remote Sensing | Topographic | | | WorldGrids |
| ACC | | Flow Accumulation | Remote Sensing | Topographic | | | HydroSheds |

**WorldClim**: http://www.worldclim.org

**CGIAR-CSI**: http://www.cgiar-csi.org

**WorldGrids**: http://worldgrids.org/doku.php

**USGS**: https://landcover.usgs.gov/green_veg.php

**GLCF**: http://glcf.umd.edu/data/treecover/data.shtml

**HydroSheds**: https://hydrosheds.cr.usgs.gov/datadownload.php
