# Supplementary material for: How many species of mammals are there in Brazil? New records of rare rodents (Rodentia: Cricetidae: Sigmodontinae) from Amazonia raise the current known diversity
Source: PeerJ. 2017 Dec 15;5:e4071. doi: 10.7717/peerj.4071 (PMC5733914; doi:10.7717/peerj.4071)
Supplement: Data S3 — List of environmental variables selected for the construction of the models to each of the three species and the importance of these variables, and for Random Forest (RF) and MaxEnt. Variable importance is represented by the mean of all model replicates, and the summed values can be different from 1. See Material and Methods section for details of variables selection. [file peerj-05-4071-s003.docx]

**How many species of mammals are there in Brazil? New records of rare rodents (Rodentia: Cricetidae: Sigmodontinae) from Amazonia raise the current known diversity**

Alexandre R Percequillo, Jeronymo Dalapicolla, Edson F Abreu Júnior, Paulo Ricardo O Roth, Katia M P M B Ferraz, Elisandra A Chiquito

**Data S3.** List of environmental variables selected after reduction of environmental autocorrelation for the construction of the models to each of the three species and the importance of these variables, and for Random Forest (RF) and MaxEnt. Variable importance is represented by the mean of all model replicates, and the summed values can be different from 1. See Material and Methods section for details of variables selection.

| **Species** | **Code** | **Description** | **Variable Importance** | |
| --- | --- | --- | --- | --- |
|  |  |  | **MaxEnt** | **RF** |
| *Neusticomys ferreirai* | BIO04 | Temperature Seasonality (standard deviation *100) | 0.64 | 0.57 |
|  | BIO18 | Precipitation of Warmest Quarter | 0.35 | 0.18 |
|  | PET07 | Global Potential Evapo-Transpiration (Global-PET) - July | 0.01 | 0.41 |
|  |  |  |  |  |
| *Neusticomys peruviensis* | BIO04 | Temperature Seasonality (standard deviation *100) | 0.38 | 0.49 |
|  | BIO18 | Precipitation of Warmest Quarter | 0.02 | 0.29 |
|  | BIO19 | Precipitation of Coldest Quarter | 0.02 | 0.06 |
|  | PET01 | Global Potential Evapo-Transpiration (Global-PET) - January | 0.36 | 0.71 |
|  | PET05 | Global Potential Evapo-Transpiration (Global-PET) - May | 0.22 | 0.10 |
|  |  |  |  |  |
| *Rhagomys longilingua* | BIO13 | Precipitation of Wettest Month | 0.02 | 0.15 |
|  | BIO16 | Precipitation of Wettest Quarter | 0.00 | 0.09 |
|  | OPISRE | Topographic Openess Index | 0.98 | 0.56 |
